# ZEB1-associated drug resistance in cancer cells is reversed by the class I HDAC inhibitor mocetinostat

**DOI:** 10.15252/emmm.201404396

**Published:** 2015-04-14

**Authors:** Simone Meidhof, Simone Brabletz, Waltraut Lehmann, Bogdan-Tiberius Preca, Kerstin Mock, Manuel Ruh, Julia Schüler, Maria Berthold, Anika Weber, Ulrike Burk, Michael Lübbert, Martin Puhr, Zoran Culig, Ulrich Wellner, Tobias Keck, Peter Bronsert, Simon Küsters, Ulrich T Hopt, Marc P Stemmler, Thomas Brabletz

**Affiliations:** 1 Department of General and Visceral Surgery, University of Freiburg Medical CenterFreiburg, Germany; 2 Spemann Graduate School of Biology and Medicine (SGBM), Albert Ludwigs University FreiburgFreiburg, Germany; 3 Faculty of Biology, Albert Ludwigs University FreiburgFreiburg, Germany; 4 Experimental Medicine I, Nikolaus-Fiebiger-Center for Molecular Medicine, FAU University Erlangen-NürnbergErlangen, Germany; 5 Oncotest GmbH, Institute for Experimental OncologyFreiburg, Germany; 6 Department of Hematology and Oncology, University of Freiburg Medical CenterFreiburg, Germany; 7 German Cancer Consortium (DKTK), Freiburg and German Cancer Research Center (DKFZ)Heidelberg, Germany; 8 Division of Experimental Urology, Innsbruck Medical UniversityInnsbruck, Austria; 9 Department of Surgery, University Medical Center Schleswig-HolsteinCampus Lübeck, Germany; 10 Tumorbank Comprehensive Cancer Center Freiburg and Institute of Surgical Pathology, University Medical Center FreiburgFreiburg, Germany

**Keywords:** cancer stem cells, drug resistance, HDAC inhibitor, miR-203, ZEB1

## Abstract

Therapy resistance is a major clinical problem in cancer medicine and crucial for disease relapse and progression. Therefore, the clinical need to overcome it, particularly for aggressive tumors such as pancreatic cancer, is very high. Aberrant activation of an epithelial–mesenchymal transition (EMT) and an associated cancer stem cell phenotype are considered a major cause of therapy resistance. Particularly, the EMT-activator ZEB1 was shown to confer stemness and resistance. We applied a systematic, stepwise strategy to interfere with ZEB1 function, aiming to overcome drug resistance. This led to the identification of both its target gene miR-203 as a major drug sensitizer and subsequently the class I HDAC inhibitor mocetinostat as epigenetic drug to interfere with ZEB1 function, restore miR-203 expression, repress stemness properties, and induce sensitivity against chemotherapy. Thereby, mocetinostat turned out to be more effective than other HDAC inhibitors, such as SAHA, indicating the relevance of the screening strategy. Our data encourage the application of mechanism-based combinations of selected epigenetic drugs with standard chemotherapy for the rational treatment of aggressive solid tumors, such as pancreatic cancer.

## Introduction

Resistance to standard radio- and chemotherapy, as well as to novel targeted therapies, is a major clinical problem in cancer medicine and crucial for disease relapse and progression. Therefore, the clinical need to overcome therapy resistance, particularly for very aggressive tumor types, such as pancreatic cancer, is very high. There are various molecular mechanisms that lead to treatment resistance, and in a general view, many of those have been linked to a stemness-associated survival phenotype (Holohan *et al*, 2013). Thus, cancer stem cells are considered to be the most resistant fraction of tumor cells, which survive different types of treatment and give rise to tumor recurrence and finally progression toward a multiresistant, often metastatic disease (Clevers, 2011; Borst, 2012). Also, the activation of an epithelial–mesenchymal transition (EMT), considered a driving force toward cancer invasion and metastasis, was associated with treatment resistance (Thiery *et al*, 2009; Floor *et al*, 2011). This is of particular relevance, since EMT and stemness were linked at molecular level, explaining why resistant cancer (stem) cells often acquired an undifferentiated EMT phenotype (Polyak & Weinberg, 2009; Singh & Settleman, 2010; Puisieux *et al*, 2014).

The EMT inducer ZEB1 is a transcriptional repressor of epithelial genes, such as E-cadherin and the miR-200 family of microRNAs. ZEB1 and miR-200 members can repress expression of each other in a double-negative feedback loop (Brabletz & Brabletz, 2010). Moreover, since miR-200 as well as miR-203, another microRNA repressed by ZEB1, can also suppress stemness traits, their downregulation by ZEB1 induces an EMT-associated stemness phenotype (Yi *et al*, 2008; Wellner *et al*, 2009). Overexpression of ZEB1, as well as subsequent downregulation of miR-200, has already been associated with a pro-survival and drug-resistant phenotype (Mongroo & Rustgi, 2010; Zhang *et al*, 2015). Furthermore, artificial re-expression of miR-200 family members has been shown to lead to a partial re-sensitization (Buck *et al*, 2007; Arumugam *et al*, 2009; Cochrane *et al*, 2009; Li *et al*, 2009; Singh *et al*, 2009; Wellner *et al*, 2009). How can this knowledge about the molecular links of EMT and drug resistance be translated to clinical application? A depletion of relevant factors, such as ZEB1, selectively in patients' cancer cells is practically impossible.

Here, we describe a systematic, stepwise approach to interfere with ZEB1 function and restore drug sensitivity by: (i) identifying additional relevant ZEB1 target genes, (ii) defining ZEB1-dependent epigenetic modifications of these genes, (iii) screening for epigenetic drugs forcing their re-expression, and (v) validating the most promising candidate drug for the restoration of treatment sensitivity. This strategy led to the detection of miR-203 as another important ZEB1 target conferring treatment sensitivity and the identification of the class I HDAC inhibitor mocetinostat, which, in contrast to other HDAC inhibitors such as SAHA, interferes with ZEB1 expression and function and restores sensitivity to chemotherapy.

## Results

### miR-203 confers drug sensitivity to ZEB1-expressing, resistant cancer cells

**Figure 1:**
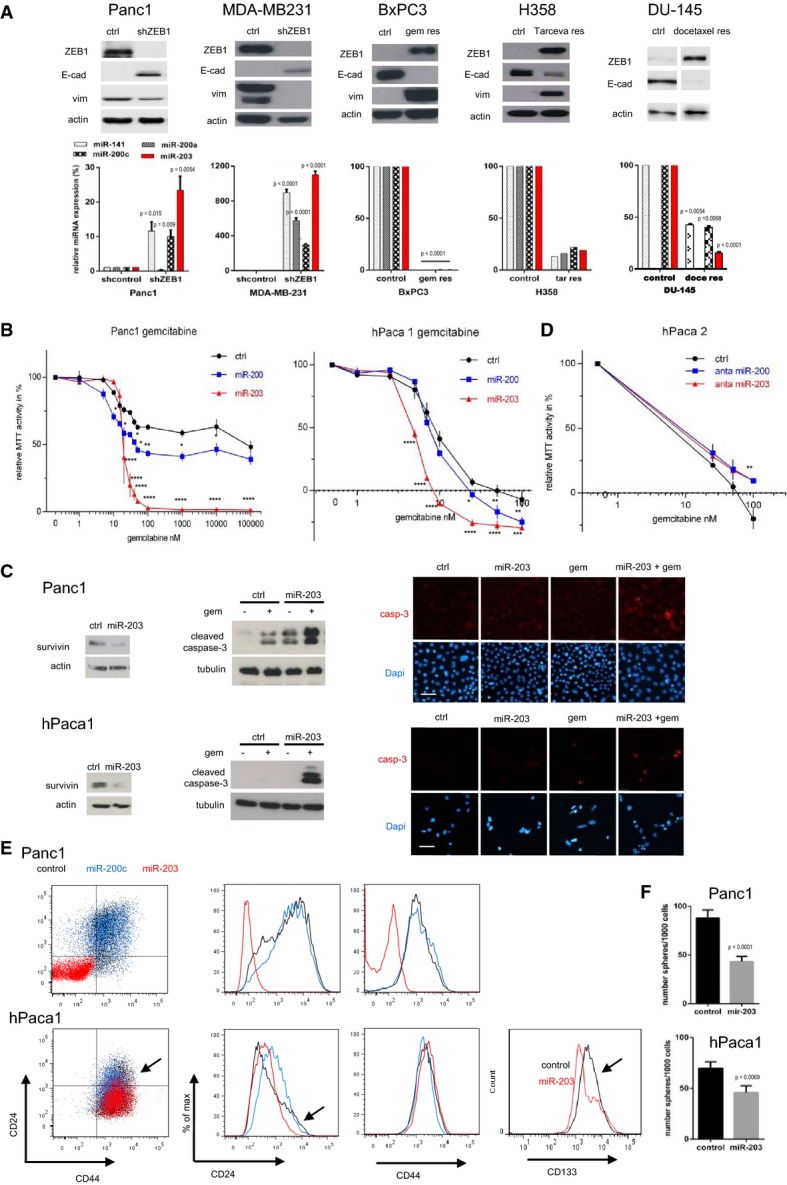
miR-203 restores drug sensitivity

EMT and, particularly, the EMT activator ZEB1 are strongly linked to a therapy resistance phenotype (Mongroo & Rustgi, 2010; Zhang *et al*, 2015). For instance, we have demonstrated that the depletion of ZEB1 in the resistant pancreatic cancer cell line Panc1 results in re-differentiation and re-sensitization to gemcitabine and that a selection for gemcitabine resistance in the sensitive pancreatic cancer cell line BxPC3 induced an EMT phenotype, with high ZEB1 and low E-cadherin expression (Wellner *et al*, 2009). The same phenotypic changes could be induced by selecting for resistance to docetaxel in the sensitive prostate cancer cell line DU145 (Puhr *et al*, 2012) and to the EGFR inhibitor Tarceva in the sensitive lung cancer line H358 (Fig1A). These data indicate that ZEB1 is a crucial determinant for mediating resistance to chemotherapeutics as well as targeted drugs in different cancer types. ZEB1 is a transcriptional repressor, and some of its major target genes, the miR-200 family, have been linked to chemosensitivity. In all cellular systems described here, ZEB1 was upregulated and miR-200 family members were downregulated in the resistant state (Fig1A). We have previously demonstrated that, like miR-200, the stemness-repressing miR-203 is also a ZEB1 target gene (Wellner *et al*, 2009), which not only suppresses stemness factors, but also anti-apoptotic factors, such as survivin and BCL-W (Bo *et al*, 2011; Bian *et al*, 2012; Wei *et al*, 2013). Moreover, like miR-200, miR-203 is also downregulated in the resistant state (Fig1A). These facts prompted us to evaluate miR-203 as a chemosensitizer. Overexpression of miR-200c increased the sensitivity to gemcitabine in the ZEB1-expressing, resistant pancreatic cancer cell lines Panc1 and MiaPaca (Fig1B and C; Supplementary Fig S1A and Table1). Strikingly, miR-203 was much more efficient than miR-200c and particularly Panc1 was sensitized to an almost complete growth inhibition. miR-203 also further sensitized the aggressive breast cancer cell line MDA-MB231 to paclitaxel, although the effect was only significant at the EC80 level (Supplementary Fig S1B and Table1).

Immunoblots and qRT–PCRs showing that expression levels of miR-203, miR-200, and E-cadherin are increased after ZEB1 knockdown in Panc1, MDA-MB-231. Vice versa, the drug-resistant clones of BxPC3, H358, and DU-145 show increased expression of ZEB1 and decreased expression of the miRNAs and E-cadherin. *n* = 3, mean ± SEM, except for H358 (data from microarray). Unpaired Student's *t*-test.

Lentiviral overexpression of miR-200c and miR-203 in Panc1 and hPaca1 induces sensitivity to gemcitabine treatment as measured by MTT assay. For the changes in EC50 values, see Table1. *n* = 3, mean ± SEM, Dunnett's multiple comparisons test (*P*-values in the graphs are *P = 0.01–0.05, **P = 0.001–0.01, ***P < 0.001, and ****P < 0.0001; for exact *P*-values, see Supplementary Table S4).

Overexpression of miR-203 decreases expression of the anti-apoptotic factor survivin and sensitizes to gemcitabine-triggered apoptosis as evaluated by cleaved caspase-3 in Western blot and immunofluorescence. Panc1 and hPaca1 were treated with 50 and 5 nM gemcitabine, respectively, for 48 h. Scale bar 20 μm.

MTT assay showing increase in gemcitabine resistance after inhibition of endogenous miRNAs in hPaca2 by specific antagomirs against miR-203 or all miR-200 members. For the changes in EC80 values, see Table1. *n* = 3, mean ± SEM, Dunnett's multiple comparisons test (*P*-values in the graphs are *P = 0.01–0.05, **P = 0.001–0.01, ***P < 0.001, and ****P < 0.0001; for exact *P*-values, see Supplementary Table S4).

Overexpression of miR-203 shows reduced numbers of the CD24/CD44 double-positive cancer stem cell population as determined by FACS analysis. The arrow indicates the reduction in the CD24 high subpopulation and reduction in CD133 by miR-203 overexpression in hPaca1 cells.

Cancer stem cell sphere assay showing reduced sphere-forming capacity of Panc1 and hPaca1 in miR-203 overexpression cells. Colonies with a diameter greater than 75 μM for Panc1 and greater 30 μM for hPaca1 cells were counted as spheres. *n* = 3, mean ± SEM, Mann–Whitney *U*-test.

Source data are available online for this figure.

**Table 1:** Shift in EC50 (EC80) by microRNAs or antagomirs

Cell line	miRNA	Drug	EC50 (nM)
Panc1	ctrl	Gemcitabine	> 10,000
	miR-200	Gemcitabine	43
	miR-203	Gemcitabine	19
MiaPaca	ctrl	Gemcitabine	830
	miR-200	Gemcitabine	22
	miR-203	Gemcitabine	23

			EC50/EC80
hPaca 1	ctrl	Gemcitabine	10.8/98
	miR-200	Gemcitabine	8.7/28
	miR-203	Gemcitabine	5.2/9.4
hPaca 2	ctrl	Gemcitabine	6.2/34
	a miR-200	Gemcitabine	8.2/88
	a miR-203	Gemcitabine	7.6/85
MDA-MB231	ctrl	Paclitaxel	4.9/1050
	miR-200	Paclitaxel	6.1/24
	miR-203	Paclitaxel	4.4/15

To validate the results in clinically more relevant settings, we isolated cancer cells from patient-derived pancreatic adenocarcinomas and selected two representative cases. hPaca1 has an undifferentiated phenotype similar to Panc1, with high ZEB1 and low E-cadherin, miR-200, and miR-203 expression, whereas hPaca2 is more differentiated with an inverse expression pattern, similar to BxPC3 (Supplementary Fig S2A). Moreover, like Panc1, hPaca1 has a CD24^high^/44^high^ subpopulation, considered to exert a tumorigenic stemness phenotype (Supplementary Fig S2B). Of note, hPaca2 is almost completely lacking this subpopulation. Compared with Panc1, both lines showed a higher, but similar sensitivity to gemcitabine (Supplementary Fig S2C). Nevertheless, the ZEB1-expressing line hPaca1 could be further sensitized to even very low gemcitabine doses by miR-203 and miR-200c overexpression. This effect was most significant at the EC80 level (Fig1B and Table1). A reverse strategy was applied for the differentiated line hPaca2, which already expresses miR-200 and miR-203. A combination of antagomirs against the endogenously expressed five miR-200 family members reduced gemcitabine sensitivity, even at high doses, again with the most significant effects reached at EC80 levels (Fig1D and Table1). Antagomir treatment against miR-203 alone had the same effect. These data underscore the role of miR-203 as inducer of drug sensitivity, which can be partially explained by a pro-apoptotic (Fig1C and Supplementary Fig S1A and B) and stemness-repressing function, as indicated by the reduction in the cancer-stem-cell-associated markers CD24^high^/44^high^ and CD133 (Fig1E), as well as the sphere-forming capacity (Fig1F). Although overexpression of miR-203 alone even enhanced the proliferative capacity, it induced (Panc1) or slightly enhanced (hPaca1) anti-proliferative effects if combined with gemcitabine (Supplementary Fig S1C).

### Identification of ZEB1-dependent epigenetic modifications on its target genes

**Figure 2:**
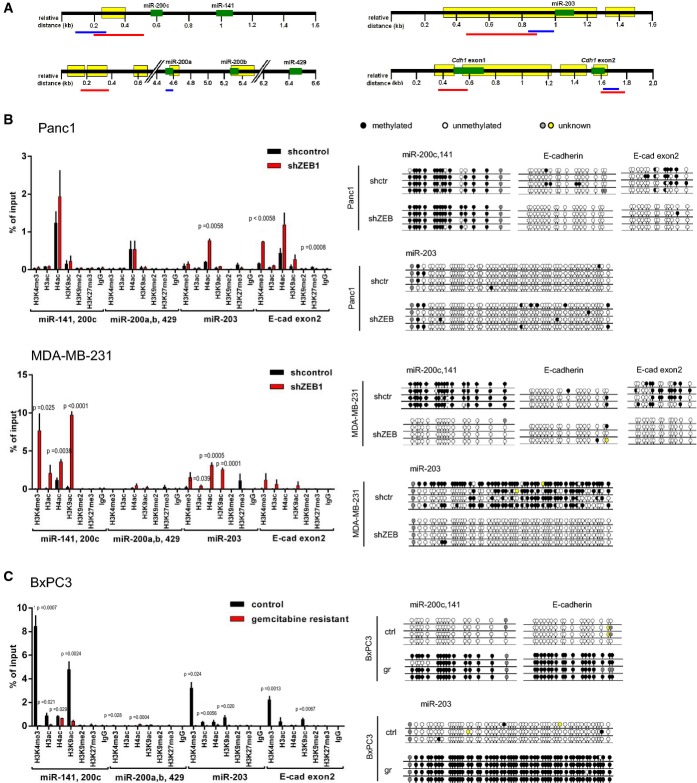
ZEB1-dependent epigenetic modifications

We aimed to interfere with ZEB1 function by forcing a re-expression of its silenced, drug-sensitizing target genes. To this end, we first determined epigenetic modifications conferred by the transcriptional repressor ZEB1 on miR-200, miR-203, and E-cadherin genes by comparing control and ZEB1 knockdown samples of the aggressive cell lines Panc1 and MDA-MB231. ZEB1 depletion induced an increase in the active histone marks H3K4me3, H3ac, H4ac, and H3K9ac in all gene loci, besides the miR-200a,b, 429 cluster (Fig2A and B). In addition, a depletion of ZEB1 resulted in a decrease in the repressive histone mark H3K27me3 on the miR-203 locus. DNA methylation patterns were also related to the ZEB1 expression status in MDA-MB231, where CpG islands in the loci of miR-203 and the miR-200c/miR-141 cluster were methylated and ZEB1 depletion resulted in an almost complete demethylation. DNA methylation patterns in Panc1 were inconsistent and not associated with the ZEB1 expression status. The ZEB1-related epigenetic changes could be verified again in the reverse setting using the drug-sensitive line BxPC3, lacking ZEB1 and expressing endogenous miR-200, miR-203, and E-cadherin (Fig2C). Here, the loci were demethylated, but the selection of drug-resistant, ZEB1-expressing clones induced a complete methylation and a reduction in active histone marks.

A Schemes for the genomic loci of the miRNA and E-cadherin genes, showing regions of the CpG islands (yellow), of the qRT–PCR amplicon for chromatin immunoprecipitation (ChIP) analysis (blue) and of the bisulfite sequencing (red).

B, C Histone marks were analyzed using ChIP coupled to qRT–PCR for Panc1 control versus shZEB, MDA-MB-231 control versus shZEB (B), and BxPC3 control versus gemcitabine resistant (gr) (C). In MDA-MB-231 and Panc1, the active histone marks H3K4me3, H3ac, H4ac, and H3K9ac were enriched. Vice versa, in the drug-resistant clones of BxPC3, the active marks were reduced in the CpG islands. The repressive histone mark H3K27me3 was not detectable in the miR-200 loci, but in the loci of miR-203 and E-cadherin in Panc1 and MDA-MB-231. DNA methylation status was determined by bisulfite sequencing. Depletion of ZEB1 in MDA-MB-231 resulted in almost complete demethylation, whereas the selection of drug-resistant, ZEB1-expressing clones in BxPC3 induced complete methylation. *n* = 2 (Panc1) or 3 (MDA-MB-231 and BxPC3), mean ± SEM; unpaired Student's *t*-test.

### Screening for epigenetic drugs interfering with ZEB1 function

We next applied a screening strategy for epigenetic drugs by selecting for their ability to re-activate expression of silenced ZEB1 target genes. Re-expression of miR-203 was used as the major readout. The best candidate(s) should then be tested for sensitizing cancer cells to chemotherapy. Based on the detected ZEB1-dependent epigenetic modifications (Fig2) as well as the known co-repressors of ZEB1 (Wang *et al*, 2007, 2009; Aghdassi *et al*, 2012; Gheldof *et al*, 2012), we focused on inhibitors of histone deacetylases (HDACs), the lysine-specific demethylase 1 (LSD1), polycomb repressor complex 2 (PRC2) factors, and DNA methyltransferases (DNMT).

**Figure 3:**
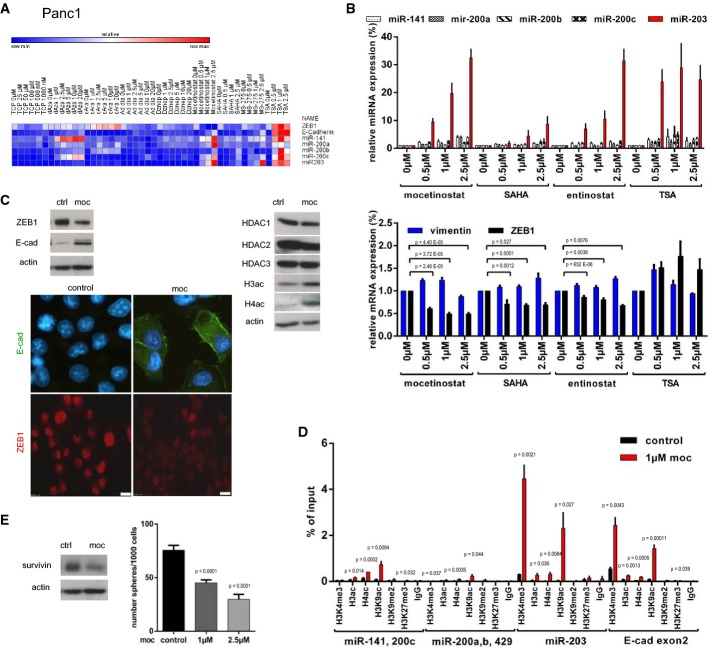
Screening of epigenetic drugs for upregulation of miRNAs and downregulation of ZEB1

Single-agent treatment with the LSD1 inhibitor TCP, the PRC2 complex inhibitors DZnep, ad dia, and cAra and the DNMT inhibitor dAza in ZEB1-expressing lines Panc1 and hPaca1 did not consistently re-activate expression of silenced miR-203 and miR-200 members (Fig3A, Supplementary Fig S2D and E and Supplementary Table S1 for statistical significance). We further concentrated on HDAC inhibitors, since HDAC1 and HDAC2 are known ZEB1 co-repressors (Wang *et al*, 2009; Aghdassi *et al*, 2012) and the most prominent ZEB1-dependent epigenetic modifications we detected are conducted by HDACs (Fig2). SAHA (vorinostat) did not or only weakly activate expression of the microRNAs (Fig3B and Supplementary Fig S2E and F). Trichostatin A, despite upregulating expression of miR-203, also led to an increase in ZEB1 in Panc1, indicating an unspecific stress reaction or gene activation induced by the applied drug doses (Fig3B and Supplementary Fig S2F). Entinostat (MS-275) and mocetinostat (MGCD0103) led to a consistent upregulation of the microRNAs, in particular of silenced miR-203. In particular, mocetinostat treatment not only strongly upregulated miR-203, but also reduced expression of ZEB1 on both mRNA and protein level (Fig3B and C, Supplementary Figs S2F and S3A). We therefore focused on this drug, which has the highest specificity for HDAC1 (Fournel *et al*, 2008). Mocetinostat induced a global increase in H3 and H4 acetylation, without changing the expression of HDACs (Fig3C and Supplementary Fig S3A) and an increase in the active histone marks H3ac, H4ac, H3K9ac, and H3K4me3 at ZEB1 target gene loci (Fig3D and Supplementary Fig S3B). Mocetinostat also conferred miR-203-related functions affecting drug resistance, such as suppression of survivin expression and suppression of stemness properties in Panc1 and hPaca1 (Fig3E and Supplementary Fig S3C). Notably, mocetinostat had no effect in the differentiated patient-derived line hPaca2, already expressing high miR-203 and low ZEB1, compared to its counterpart hPaca1 (Supplementary Fig S3A).

Heat map showing the relative expression levels after drug treatment for 48 h in Panc1. Values measured by qRT–PCR were depicted with the software GENE-E. Only mocetinostat upregulated the miRNAs and downregulated ZEB1.

Relative expression of indicated genes in Panc1 measured by qRT–PCR after treatment with different HDAC inhibitors. Note the downregulation of ZEB1 and upregulation of miR-203, miR-200, and E-cadherin by mocetinostat. *n* = 3, mean ± SEM; unpaired Student's *t*-test. For significance, see Supplementary Table S1.

Immunoblot and immunofluorescence showing that mocetinostat treatment (1 μM, 48 h) reduced ZEB1 expression and induced E-cadherin in Panc1. Expression of histone deacetylases was not altered by mocetinostat, but histone acetylation was induced. Scale bar 10 μm.

Chromatin immunoprecipitation analysis validated mocetinostat-induced (1 μM, 48 h) enrichment of the active histone marks H3ac, H4ac, H3K9ac, and H3K4me3 at ZEB1 target gene loci in Panc1. *n* = 3, mean ± SEM; unpaired Student's *t*-test.

Mocetinostat treatment reduced expression of the anti-apoptotic miR-203 target survivin and sphere-forming capacity in Panc1 when pre-treated with mocetinostat for 48 h. *n* = 3, mean ± SEM; Mann–Whitney *U*-test.

Source data are available online for this figure.

### The class I HDAC inhibitor mocetinostat restores drug sensitivity

**Figure 4:**
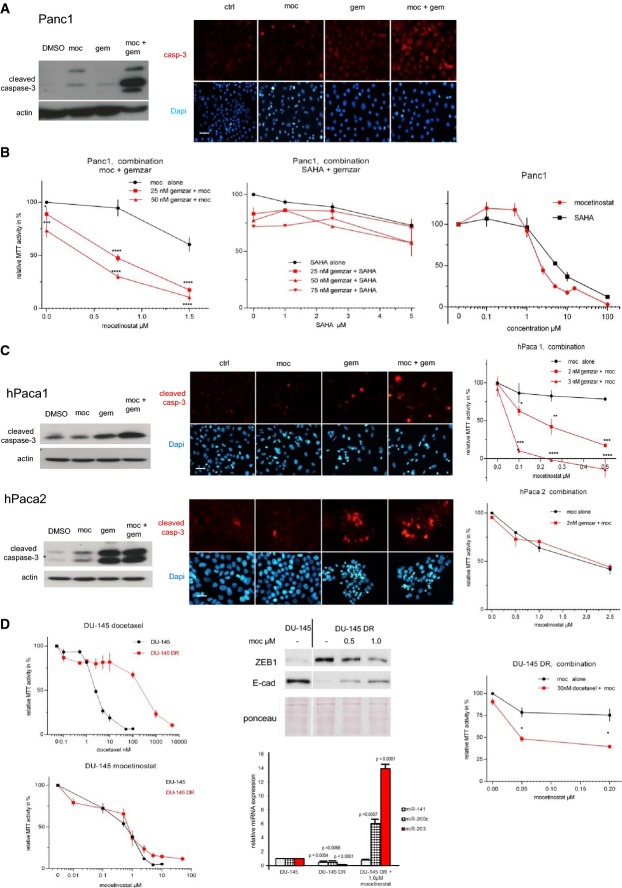
Mocetinostat sensitizes to gemcitabine *in vitro*

The effects of mocetinostat on two regulators of drug resistance—downregulation of ZEB1 expression and upregulation of miR-203—prompted us to investigate whether this substance can restore drug sensitivity. We now largely focused on pancreatic cancer, since this tumor has a particular poor prognosis and treatment options are rare. Gemcitabine treatment alone did not induce apoptosis in the resistant line Panc1. However, mocetinostat, which alone only had a weak pro-apoptotic effect, sensitized Panc1 cells for gemcitabine-induced apoptosis (Fig4A) and a dose-dependent growth inhibition by gemcitabine (Fig4B and Table2). A sensitizing effect of mocetinostat was also detected in the patient-derived line hPaca1, expressing high ZEB1 and low miR-203 (Fig4C and Table2). Strikingly, mocetinostat had no further drug-sensitizing effect in hPaca2 with already high endogenous miR-203 and miR-200, and low ZEB1 (Fig4C and Table2), indicating that if ZEB1 is not expressed and both microRNAs are already present, mocetinostat is less effective in sensitizing to gemcitabine. Supporting the efficiency of our screening strategy, SAHA, which did not or only weakly re-activate expression of miR-203 or downregulate ZEB1 (Supplementary Fig S2E and F), had no drug-sensitizing effect, although its inhibitory effect as single agent was similar to mocetinostat (Fig4B and Table2). We further wanted to know whether the drug-sensitizing effect of mocetinostat is depending on an upregulation of miR-200 or miR-203. We were not able to proof a direct mechanistic link between the drug-sensitizing effect of mocetinostat and the upregulation of miR-203 and miR-200, although a combined inhibition of miR-203 and miR-200 by antagomirs in mocetinostat- and gemcitabine-treated cells led to an increase in MTT activity, which would fit to the hypothesis. But the result was not informative, since antagomir treatment also increased MTT activity of gemcitabine-treated cells (Supplementary Fig S3D).

Immunoblot and immunofluorescence for cleaved caspase-3 showing strong increase in apoptosis in gemcitabine-resistant Panc1 after combined treatment with mocetinostat (1 μM) and gemcitabine (50 nM). Scale bar 20 μm.

MTT assay for Panc1 treated with the indicated concentrations of mocetinostat (left) or SAHA (middle) and gemcitabine (72 h). Combined treatment of mocetinostat and gemcitabine significantly reduced cell viability. In contrast, a combination with SAHA had no effect. For calculation of the CI and synergy between the drugs, see Table2. Comparison of mocetinostat and SAHA alone (right). *n* = 3, mean ± SEM, Dunnett's multiple comparisons test (*P*-values in the graphs are ***P < 0.001 and ****P < 0.0001; for exact *P*-values, see Supplementary Table S4).

Effects of mocetinostat on cleaved caspase expression and susceptibility to gemcitabine in the patient-derived pancreatic cancer cells. Note that hPaca1 behaves similar to Panc1, but mocetinostat had no significant effect in hPaca2. Scale bar 20 μm. *n* = 3, mean ± SEM, Dunnett's multiple comparisons test (*P*-values in the graphs are *P = 0.01–0.05, **P = 0.001–0.01, ***P < 0.001, and ****P < 0.0001; for exact *P*-values, see Supplementary Table S4).

MTT assays comparing the effects of docetaxel and mocetinostat in the prostate cancer cell line DU-145 and the docetaxel-resistant subclone DU-145 DR (left). Mocetinostat treatment of DU-145 DR downregulates ZEB1, upregulates E-cadherin, miR-200, and miR-203 expression. For relative miRNA expression, the expression levels in original DU-145 were set to 1 (middle panels, the immunoblot panel derives from the same experiment shown in Fig1A). Mocetinostat sensitizes DU-145 DR to docetaxel (right). For calculation of the CI and synergy between the drugs, see Table2. *n* = 3, mean ± SEM, Dunnett's multiple comparisons test (*P*-values in the graphs are *P = 0.01–0.05; for exact *P*-values, see Supplementary Table S4).

Source data are available online for this figure.

**Table 2:** Effects of combinations of mocetinostat or SAHA with chemotherapeutics

Cell line	Moc plus	Gemzar (nM)	EC50 moc (μM)	CI	Effect
Panc1		0	2.4		
		25	0.71	0.29	Synergy
		50	0.41	0.17	Synergy
hPaca 1		0	0.68		
		2	0.19	0.47	Synergy
		3	0.07	0.41	Synergy
hPaca 2		0	2.2		
		2	2.4	> 1	No

		Docetaxel (nM)			
DU145 DR		0	0.62		
		30	0.05	0.21	Synergy

CI = combination index; < 1 = synergistic.

The effects of mocetinostat could also be validated in a different cancer type treated with a different type of chemotherapeutic. We used a resistant subclone (DR-145 DR) of the prostate cancer cell line DU-145, which is normally highly sensitive to the taxol-derivate docetaxel. Also, the resistant DU-145 DR underwent an EMT with the upregulation of ZEB1 and downregulation of E-cad, miR-200, and miR-203 (Puhr *et al*, 2012) (Fig1A). Although showing strong difference in EC50 to docetaxel, both clones had a similar sensitivity to mocetinostat as a single agent (Fig4D). As demonstrated for the ZEB1-expressing pancreatic cell lines, mocetinostat treatment of DR145-DR also partially reversed the EMT phenotype, downregulated ZEB1 expression, upregulated miR-200c and miR-203, and restored chemosensitivity in a synergistic manner, in this case to the taxol-derivate docetaxel (Fig4D and Table2).

**Figure 5:**
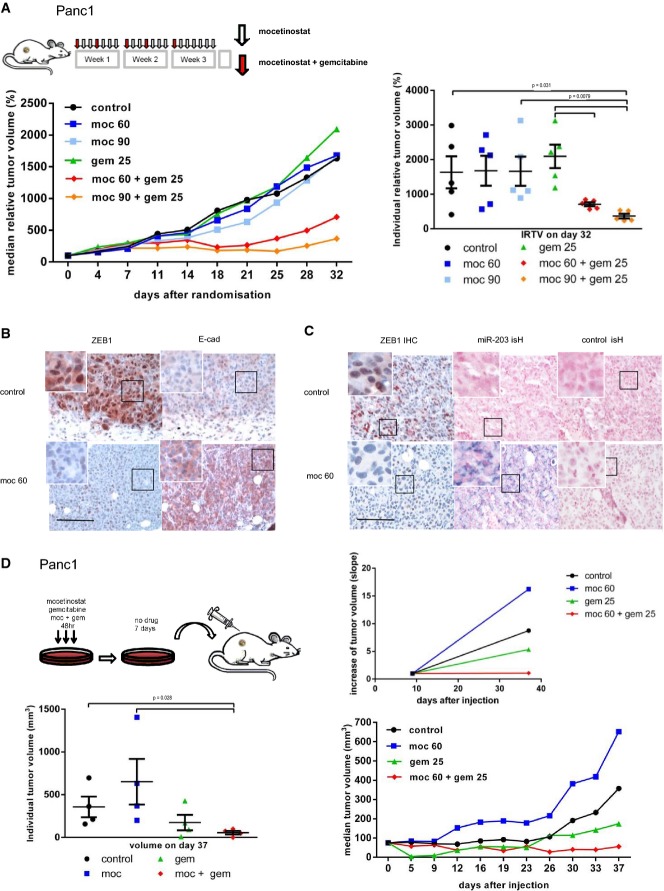
Mocetinostat sensitizes to gemcitabine *in vivo*

The *in vitro* results were validated in *in vivo* xenograft studies. Mocetinostat treatment led to a dose-dependent increase in the sensitivity of Panc1-derived tumors to gemcitabine, whereas single application of either drug had no significant tumor-inhibiting effect or even increased tumor growth (Fig5A). For hPaca1, a combination of gemcitabine with mocetinostat also strongly reduced tumor growth, although in this case, the single treatment already had a tumor-inhibiting effect (Supplementary Fig S4A). Expression analyses of the tumors further documented the effect of mocetinostat. Consistent with the results in cell culture, mocetinostat induced a downregulation of ZEB1 expression and an upregulation of E-cadherin and miR-203 in both Panc1- and hPaca1-derived tumors (Fig5B and C and Supplementary Fig S4B and C). As observed in cell culture experiments, mocetinostat did not change the *in vivo* phenotype and *in vivo* sensitivity of hPaca2 (Supplementary Fig S4D and E).

Relative tumor volume (RTV) of Panc1-derived tumors in NMRI nu/nu mice. Eleven days after implantation, mice were randomized according to tumor volume. Treatment with mocetinostat (60 or 90 mg/kg/day) and gemcitabine (25 mg/kg/day) was implemented (day 0) as depicted in the scheme. Shown are the group medians of the RTVs over time (left) and the individual RTVs on day 32 (right). *n* = 5 for each treatment group; nonparametric Mann–Whitney *U*-test.

Representative immunohistochemical stainings of serial sections showing reduced ZEB1 and increased E-cadherin in tumor tissues of mice treated with mocetinostat. Scale bar 40 μm, inserts for higher magnifications 10 μm. Squares indicate magnified regions.

Representative pictures of *in situ* hybridization for miR-203 and control probe showing gain of miR-203 and associated loss of ZEB1 detected by immunohistochemistry in serial sections of mocetinostat-treated xenograft tumors. Scale bar 40 μm, inserts for higher magnifications 5 μm. Squares indicate magnified regions.

Schematic outline and results for xenografts of *ex vivo* treated Panc1 in Foxn1 nude mice. Panc1 cells were pre-treated with mocetinostat (1 μM) and/or gemcitabine (50 nM) for 48 h, followed by a 7-day recovery period before being injected subcutaneously (left). Equal numbers of viable cells were injected in 75 μl volume. At day 9 after injection, tumor growth was detectable in all groups (lower right). To better visualize and compare tumor growth, the tumor volume at day 9 was set to 1 and the increasing slope of the tumor volume to day 37 is depicted (upper right). The individual absolute tumor volumes on day 37 (lower left) and the group medians of the absolute tumor volumes over time (lower right) are shown. For cells pre-treated with the combination of mocetinostat and gemcitabine, tumor growth was arrested. *n* = 4 for each treatment group; nonparametric Mann–Whitney *U*-test.

Mocetinostat has been described to possess a long-lasting effect *in vitro* upon drug removal (Fournel *et al*, 2008). To test this effect *in vivo*, Panc1 cells were transiently exposed *ex vivo* for 48 h to mocetinostat and/or gemcitabine, followed by 7 days of drug withdrawal and subsequent xenografting of identical numbers of viable cells. Gemcitabine pre-treatment alone reduced tumor growth. Interestingly, mocetinostat pre-treatment alone even enhanced tumor growth, but when combined with gemcitabine further sensitized for growth inhibition (Fig5D). These data indicate that the effect of mocetinostat persists for extended time periods and is not an immediate cytotoxic effect, but rather based on sustained changes in chromatin structure and gene expression.

### Clinical relevance

**Figure 6:**
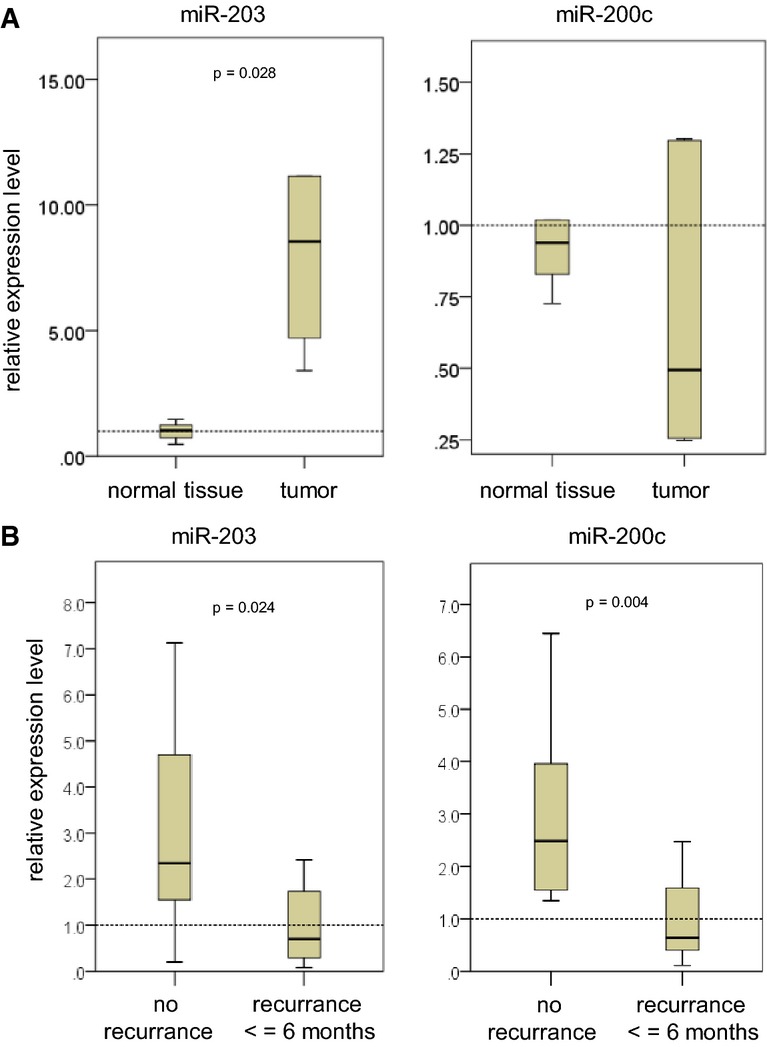
Clinical relevance of miR-203 expression

Our preclinical data indicate that patients with aggressive, highly resistant cancers might benefit from such combination therapies. However, a validation of the underlying mechanisms also in patients' tumors would be required. In a small-scale pilot study, we determined whether miR-203 expression in pancreatic cancers correlates with clinical outcome. We selected cases, which underwent curative surgery (R0 resection) and adjuvant gemcitabine treatment, and stratified them in two groups, with no recurrence after more than 2 years and early recurrence within 6 months. Interestingly, in contrast to miR-200c, miR-203 was upregulated in pancreatic adenocarcinomas compared with normal pancreatic tissue (mean relation normal versus tumor: miR-203 ×7.8; miR-200c ×0.68) (Fig6A and Supplementary Table S2). However, the expression of both miRNAs in pancreatic cancers was heterogenous and the expression level differed between the two groups. The non-recurrence group showed a significant association with high expression of miR-203 and miR-200c compared to the recurrence group (mean relation non-recurrence versus recurrence: miR-203 ×2.79; miR-200c ×3.53), which might indicate an increased benefit of gemcitabine treatment in cases with high miR-203 levels (Fig6B and Supplementary Table S3).

A, B Relative expression levels of miR-203 and miR-200c in pancreatic adenocarcinomas. (A) In normal versus tumor tissue of the same case (*n* = 6 cases), (B) in the non-recurrence (*n* = 10 cases) versus the recurrence group (*n* = 11 cases). The mean value of the lower group in each figure was set to 1. Nonparametric Mann–Whitney *U*-test.

## Discussion

Here, we describe a chain of molecular events important for cancer treatment resistance and the development of specific strategies to overcome it. Given the role of ZEB1 in mediating resistance to different types of cancer drugs, overexpression of one of its major target genes, miR-203, can efficiently restore drug sensitivity. Using re-expression of silenced miR-203 as readout, we applied a screening strategy for epigenetic drugs and identified the HDAC class I-specific inhibitor mocetinostat as a promising candidate. The efficiency of this strategy was validated particularly for pancreatic cancer, by demonstrating that mocetinostat reduced ZEB1 expression, upregulated expression of miR-203 and other ZEB1 targets, and sensitized undifferentiated, ZEB1-expressing cancer cells for chemotherapy.

The mechanisms underlying ZEB1-associated treatment resistance are complex and likely also include unknown, target gene independent effects. However, our data indicate that besides miR-200, the ZEB1 target miR-203 is an important factor. miR-203 has been described as a stemness-inhibiting microRNA (Lena *et al*, 2008; Yi *et al*, 2008; Taube *et al*, 2013), by suppressing factors such as the self-renewal factor Bmi1 (Shimono *et al*, 2009; Wellner *et al*, 2009). Since stemness properties are considered to confer a survival phenotype, miR-203 might exert a dual effect to restore drug sensitivity: by inhibiting stemness and by directly favoring apoptosis. Although shown to be directly pro-apoptotic by targeting the anti-apoptotic factors survivin (Wang *et al*, 2012; Wei *et al*, 2013) and BCL-W (Bo *et al*, 2011), its function concerning drug resistance is inconsistent. It has been shown to increase sensitivity of leukemic cells to ATO (He *et al*, 2013b), lung cancer cells to gefitinib (Garofalo *et al*, 2012), and colon cancer cells to paclitaxel (Li *et al*, 2011), but also to suppress sensitivity of breast cancer cells to cisplatin (Ru *et al*, 2011). Our data support a drug-sensitizing effect of miR-203. Inconsistent data are also found for the association of miR-203 expression with clinical outcome: High expression indicated good prognosis in glioma (He *et al*, 2013a) and prostate (Saini *et al*, 2011), but surprisingly poor prognosis in colon (Bovell *et al*, 2013) and pancreatic cancer (Greither *et al*, 2010; Ikenaga *et al*, 2010). Our data link high expression of miR-203—and low expression of ZEB1—to reduced tumor recurrence and metachronous metastasis after curative surgery and adjuvant chemotherapy. A potential explanation for the conflicting data could be that miR-203 acts as a double-edged sword: It confers both growth advantage and treatment sensitivity by reducing the resistant and potentially low-cycling cancer stem cell fraction. This hypothesis is in line with our data that overexpression of miR-203 alone can increase proliferation (Supplementary Fig S1C) and that it is upregulated in pancreatic cancers compared with normal pancreas (Fig6A). Furthermore, our data comparing patient-derived cancer cells indicate that high ZEB1/low miR-203 expression might predict a favorable effect of mocetinostat in sensitizing cancer cells. Ongoing analyses on larger patient cohorts will show whether miR-203 is a predictive marker to stratify pancreatic adenocarcinomas (PDACs) in treatment-sensitive and treatment-resistant subgroups. This might also include the classification into newly described PDAC subtypes (Collisson *et al*, 2011).

Upregulation of miR-203 turned out to be an efficient readout to screen for chemosensitizing substances, leading to the identification of the HDACi mocetinostat as a candidate. Notably, mocetinostat not only induced re-expression of relevant ZEB1 targets, but also led to a reduction in ZEB1 itself, probably indirectly on protein level through translational suppression by re-expressed miR-200 members (Brabletz & Brabletz, 2010) and directly by affecting its transcription, since the ZEB1 promoter has a bivalent chromatin configuration, allowing for a very dynamic adaptation (Chaffer Christine *et al*, 2013). As a positive consequence, additional unknown mechanisms concerning ZEB1-mediated resistance are also targeted by mocetinostat. A drug-sensitizing effect of mocetinostat has already been described (Sung *et al*, 2011). Here, we demonstrated it to be specific compared to other HDACis, for example, SAHA, and described the selective downregulation of ZEB1 and upregulation of its target genes by mocetinostat as one potential mechanism, which strongly correlated with differences of mocetinostat and SAHA in sensitizing to gemcitabine. However, tests proofing a direct mechanistic link between the drug-sensitizing effect of mocetinostat and the upregulation of miR-203 or miR-200 did not reach significance levels (Supplementary Fig S3D). Therefore, we could not make an unambiguous statement concerning the crucial downstream effectors of mocetinostat. As HDAC inhibitor, it likely has many, still unknown molecular effects and target genes with potential relevance for its efficiency in restoring drug sensitivity and we do not want to claim that the upregulation of miR-203/200 and inhibition of ZEB1 are its only or major effects in this context.

What could be the molecular basis for stronger effect of mocetinostat versus other HDACis, in particular SAHA, in downregulating ZEB1 and upregulating its target genes? ZEB1 has been shown to interact with HDACs1/2 (Wang *et al*, 2009; Aghdassi *et al*, 2012), and we detected strongest differences in the histone marks H3ac, H4ac, and H3K9ac, which are conferred by these enzymes, both after changes in ZEB1 expression and mocetinostat treatment. Compared to other HDACis such as SAHA, mocetinostat is highly specific for HDAC1 versus HDACs 2, 3, 11 and inert to HDACs 4, 5, 6, 7, 8 (Fournel *et al*, 2008), which might explain a more selective and stronger effect. Also, the increase in the active mark H3K4me3 on ZEB1 target genes after mocetinostat treatment (Fig3D and Supplementary Fig S3B), which is attributable to the ability of class I HDACis to repress the JARID1 family of histone H3 lysine 4 demethylases (Huang *et al*, 2011), can add to the effect of this HDACi. As described for the miR-200c/miR-141 gene cluster, DNA methylation and HDAC-mediated histone marks depend on the EMT state (Davalos *et al*, 2011; Lim *et al*, 2013). We also found that ZEB1-dependant changes in the EMT state induced the most prominent changes for HDAC-dependent marks. In contrast, although ZEB1 is considered a transcriptional repressor, we detected no significant changes for repressive histone marks H3K9me2 and H3K27me3 conferred by its cofactor LSD1 and PRC2 complex factors, explaining lower efficacy of their inhibitors in restoring the expression of miR-203. Our findings that even members of a restricted drug subgroup (here HDACis) show very different efficiency further underscore the relevance of the accurate screening strategy, as exemplified here with miR-203 re-expression as successful readout system.

In conclusion, our strategy aimed to use epigenetic drugs to restore chemosensitivity by inducing differentiation of resistant cancer cells, which are otherwise trapped in an EMT/stemness phenotype. Epigenetic drugs are of particular relevance, since they can effectively induce a long-term antitumor memory response, even at low doses without an immediate cytotoxic effect (Baylin & Jones, 2011; Tsai *et al*, 2012). Our data obtained from *in vivo* experiments using cancer cells pre-treated with mocetinostat (Fig5D) support these findings. A clinical trial using a combination of low-dose DNMT inhibitors and doxorubicin has already been successful for the treatment of diffuse large B-cell lymphoma (Clozel *et al*, 2013). Our results encourage the application of rational, mechanism-based combinations of selected epigenetic drugs and standard chemotherapy also for the treatment of aggressive solid cancers, such as subtypes of pancreatic cancer.

## Materials and Methods

### Cell culture and drug treatment

All cell lines were purchased from ATCC and cultured according to the instructions. BxPC3 gemcitabine-resistant cells were established previously (Wellner *et al*, 2009). Accordingly, Tarceva-resistant H358 cells were selected by culturing for several weeks in DMEM/10% FCS containing gradually increasing concentrations of Tarceva (1 μM to 5 μM) (OSI Pharmaceuticals). Generation and characterization of docetaxel-resistant subclones of the prostate cancer cell DU-145 were previously described (Puhr *et al*, 2012). Mocetinostat/MGCD0103 (Chemietek), gemcitabine (Sigma), vorinostat/SAHA (Selleck), trichostatin A/TSA (Selleck), entinostat/MS-275 (Selleck), trans-2-phenylcyclopropyl-amine hydrochloride/TCP (Sigma), 3-deazaneplanocin A/DZnep (Sigma), cytosine β-D-arabinose/cAra (Sigma), adenosine dialdehyde/ad dia (Sigma), 5′-aza-2′deoxycytidine/dAza (Sigma), or mock were added for 48 h, in MTT assays for 72 h. Cells were then harvested for specific assays. The heat map for the drug-treated cells was created with the software GENE-E (Broad Institute). Stable clones for shZEB1 and shcontrol knockdown were established as previously described (Spaderna *et al*, 2008). For generation of clones stably overexpressing miR-200c or miR-203, lentiviral expression vectors were produced and cells were infected as previously described (Brabletz *et al*, 2011), followed by selection under standard conditions in DMEM/10% FCS + 2 μg/ml puromycin. The absolute expression of overexpressed miRNAs was at comparable levels as determined by qRT–PCR.

### Isolation of human patient-derived pancreatic cancer cells and tissue specimen

Human tissues and tumor cells from human pancreatic cancer were obtained with patients' consent, as approved by the Ethics Commission of the University Freiburg Medical Center (no. 13/11,130538). Isolation of patient-derived cancer cells was done as previously described (Smith *et al*, 2008). In brief, patients' tumor explants were cut into small pieces (2 mm^3^), which were further implanted subcutaneously into 7-week-old NMRI nu/nu female mice. After in-mouse passaging, viable human pancreatic tumor cells were isolated, characterized as described, tested for mycoplasma contamination, and used for further assays. hPaca2 was isolated from a well-differentiated pancreatic adenocarcinoma and hPaca1 from a moderately differentiated pancreatic adenocarcinoma. Formalin-fixed, paraffin-embedded samples of pancreatic carcinomas and associated clinical follow-up data from patients, who underwent curative surgery (R0) and adjuvant chemotherapy with gemcitabine, were retrieved from the University Freiburg Medical Center. Tumor tissue was isolated by microdissection. RNA isolation and quantification of miR-200c and miR-203 expression in tumors were performed as previously described (Brabletz *et al,* 2011).

### Microarrays

The microarray data from this publication have been submitted to the ArrayExpress database (https://www.ebi.ac.uk/arrayexpress/) and assigned the identifiers E-MTAB-3387 and E-MTAB-3391.

### Specific inhibition of miRNAs using antagomirs

Antagomirs (Dharmacon) were designed as described (Krutzfeldt *et al*, 2007). A total of 3 μM antagomirs were added to the normal cell culture medium right after seeding in 6-well plates. Cells were harvested for specific assays 3–4 days later.

### Cell viability assay (MTT)

All cell lines except hPaca1 were seeded with 3,000 cells per well in 96-well format. hPaca1 was seeded in 12-well plates with 50,000 cells per well. After 24 h, cells were treated as indicated. After 72 h, 5 mg/ml MTT (methylthiazolyldiphenyl-tetrazolium bromide; Sigma) was added to the medium and incubated for 4 h. The medium was removed and precipitates were dissolved in 200 μl acidified isopropanol (0.04 N HCl). Absorption was measured at 570 nm with 650 nm as a reference wave length. Relative MTT activity was then calculated relative to activity 1 day after seeding (set to 0%). The activity of untreated cells 72 h after starting of drug treatment was set to 100%. A negative activity means that the drug reduced cell number below the number of cells at treatment start. Significance in differences was calculated with the two-way ANOVA, Dunnett's multiple comparisons test (*P*-values in the graphs are: *P = 0.01–0.05, **P = 0.001–0.01, ***P < 0.001, ****P < 0.0001; for exact *P*-values see Supplementary Table S4).

### BrdU incorporation

A total of 3,000 cells/well for Panc1 and 1,500 cells/well for hPaca1 were seeded in 96-well plates. BrdU was added for a 4-h pulse and incorporation measured by ELISA at 450 nm (BrdU Cell Proliferation Assay Kit, Cell Signaling Technology) according to the manufacturer's instructions. To test for inhibition of proliferation by gemcitabine, 24 h after seeding, cells were treated with gemcitabine and, 72 h after beginning treatment, BrdU was added for 4 h.

### Bisulfite sequencing

Genomic DNA was extracted using QIAamp DNA Mini Kit (Qiagen) and bisulfite-treated with the EZ DNA methylation kit (Zymo Research) according to the manufacturer's instructions. Following PCR amplification of the bisulfite-converted DNA, the product was cloned using the TOPO TA Cloning Kit (Invitrogen) and sequenced. DNA methylation status was analyzed with the CpG viewer software.

### Cancer stem cell spheroid assay

For the detection of cancer stem cell potential, cell lines were processed in sphere-forming assay as described previously (Wellner *et al*, 2009). The number of colonies with a diameter > 75 μm for Panc1 and > 30 μm for hPaca1 was counted after 7 days.

### Immunoblots

Western blots were performed as previously described (Spaderna *et al*, 2008). A total of 40 μg of protein was separated by SDS–PAGE, transferred to nitrocellulose membrane, and immunoblotted using rabbit anti-ZEB1 (1:5,000, HPA027524; Sigma), rabbit anti-cleaved caspase-3 (1:1,000, 9664; Cell Signaling), rabbit anti-LC3BII (1:1,000, 3868; New England Biolabs), rabbit anti-survivin (1:1,000, NB500-201; Novus), mouse anti-E-cadherin (1:1,000, 610182; BD Biosciences), mouse anti-vimentin (1:1,000, M0725; Dako), rabbit anti-H3ac (1:2,000, 06-599; Millipore), rabbit anti-H4ac (1:1,000, 06-598; Millipore), and mouse anti-actin (1:5,000, A5441; Sigma) or mouse anti-tubulin (1:5,000, T6199, Sigma) to control loading efficiency.

### RNA isolation and quantitative RT–PCR

RNA was isolated using RNeasy Plus Mini Kit (Qiagen). For microRNA quantification, cDNA was synthesized with the miRCURY LNA cDNA synthesis kit (Exiqon) using 1,000 ng of RNA as template and diluted 1:60. For mRNA, cDNA was synthesized with the RevertAid First Strand cDNA Synthesis Kit (Thermo Scientific) according to the manufacturer's instructions. Expression values were measured in triplicate on a Roche LightCycler 480 and normalized to β-actin expression (mRNA) and to miR-16 (microRNA). Results are computed as fold induction relative to controls.

### Chromatin immunoprecipitation of histone modifications

Chromatin immunoprecipitation (ChIP) was performed as previously described (Wohrle *et al*, 2007) with the following modifications: Samples were sonicated for 30 min with 30-s intervals on power level ‘high’ with the Diagnode Bioruptor UCD200. The optical density at 260 nm was determined, and aliquots corresponding to 50 optical density units were used for each IP. The lysates were diluted with two sample volumes of IP dilution buffer before adding 5 μg of appropriate antibodies and 25 μl of Protein G magnetic beads (Active Motif). The antibodies rabbit anti-acetyl histone H3 (06-599), rabbit anti-acetyl histone H4 (06-598), rabbit anti-trimethyl histone H3K4 (04-745), anti-acetyl histone H3K9 (17-658), rabbit anti-trimethyl histone H3K27 (07-449), rabbit anti-dimethyl histone H3K9 (17-648), and isotype control immunoglobulin G (IgGs, PP64B) were purchased from Millipore. For qRT–PCR, 2.5 μl of the immunoprecipitated DNA and 2% of the reference material were used as templates.

### Immunofluorescence

Cells were fixed with 4% formaldehyde or ice-cold methanol and blocked with PBS/2% normal goat serum. Fixed cells were incubated with the primary antibodies mouse anti-E-cadherin (1:100, 610182; BD Biosciences), rabbit anti-ZEB1 (1:200, HPA027524; Sigma), rabbit anti-cleaved caspase-3 (1:400, 9664; Cell Signaling), rabbit anti-LC3BII (1:100, 3868; New England Biolabs), and mouse anti-vimentin (1:500, M0725; Dako) as indicated in the text at 4°C overnight, followed by Alexa Fluor® 488-conjugated goat anti-mouse IgG (1:500, A-11029; Life Technologies) and Cy3®-conjugated goat anti-rabbit IgG (H+L) (1:250, A10520; Life Technologies) or Alexa Fluor® 488-conjugated goat anti-rabbit IgG (1:500, A-11034; Life Technologies) and Cy3®-conjugated goat anti-mouse IgG (H + L) (1:250, A10521; Life Technologies) for 1 h at room temperature and counterstained with DAPI (Molecular Probes).

### Fluorescence-activated cell sorting (FACS) analysis

For FACS analysis, cells were collected with 0.05% trypsin–EDTA solution, washed, and diluted to 1 million cells per ml in PBS/2% FCS containing the diluted primary antibodies anti CD24-PE (555428; BD Bioscience) and anti CD44-APC (561862; BD Bioscience) or anti CD133 (AC133, PE,130-080-801; Miltenyi Biotec) incubated in the dark for 20 min at room temperature, washed with PBS/2% FCS, resuspended in PBS/2% FCS at 0.5–1 million cells per ml, and analyzed using a BD LSR Fortessa and BD FACSDiva Software (Becton Dickinson). A total of 10,000 viable cells were counted. Dot plots and histograms were generated with FlowJo software.

### Xenograft *in vivo* assays

All animal experiments were performed in accordance with Animal Welfare and approved by the local authorities (no. G-12/44). Pre-established fresh frozen fragments (3 mm^3^) of tumors derived from xenografted Panc1, hPaca1, or hPaca2 were implanted subcutaneously into 7-week-old NMRI nu/nu female mice. After 11 days (Panc1), 18 days (hPaca1), or 10 days (hPaca2), mice were randomized according to tumor volume into the different treatment groups and received single treatment or combinations of i.v. injections of gemcitabine- and p.o.-administered mocetinostat or vehicle control at the indicated concentrations and time points. Five mice were used in each treatment group. Tumors were measured twice weekly, and relative tumor volume (RTV) was calculated by setting the volume of the individual tumor at the day of randomization to 100%. Mice were sacrificed at indicated time points and tumors were formalin-fixed and paraffin-embedded. For *in vivo* analyses of pre-treated tumor cells, Panc1 cells were treated with 1 μM mocetinostat, 50 nM gemcitabine, a combination of both, or vehicle (DMSO) for 48 h, followed by another 7 days in culture without drug. A total of 2 million viable cells in a volume of 75 μl were injected subcutaneously into the flank of 5-week-old Foxn1 nude female mice (Harlan). Four mice were used in each treatment group. Tumors were measured twice weekly and absolute tumor volume was calculated. On day 9 after injections, tumors in all treatment groups were palpable. In order to better visualize and compare the tumor growth, the tumor volume at day 9 was set to 1 and the increasing slope of the tumor volume to day 37 was shown. At the end of the experiment, mice were sacrificed.

### Immunohistochemistry

Immunohistochemistry on formalin-fixed, paraffin-embedded samples of mouse xenografts was performed as previously described (Brabletz *et al*, 2004), using rabbit anti-ZEB1 (1:200, HPA027524; Sigma) and mouse anti-E-cadherin (1:100, 610182; BD Biosciences) antibodies.

### *In situ* Hybridization

Formalin-fixed, paraffin-embedded tissues were sectioned at 6 μm, deparaffinized, rehydrated, and treated with proteinase K (15 μg/ml, 20 min, 37°C). Slides were formaldehyde- and EDC-fixed and then acetylated as described previously (Pena *et al*, 2009). Sections were dehydrated and hybridized overnight at 50°C with 80 nM miRCURY LNA detection 5′-DIG and 3′-DIG-labeled probe for miR-203 and scrambled control (Exiqon). LNA U6 snRNA (Exiqon) was used as a positive control at 10 nM. After hybridization, further steps were performed using the instruction of v2.0 miRCURY LNA microRNA ISH Optimization Kit for FFPE (Exiqon).

### Statistical analysis

Statistical evaluation was performed using GraphPad Prism 6. Each experiment was repeated three times or more, unless otherwise indicated. Data are presented as mean ± SEM. For qRT–PCR analysis, unpaired Student's *t*-test was used to compare two groups of independent samples and a normal distribution was assumed. For the spheroid assay and the *in vivo* experiments, Mann–Whitney *U*-tests were used. For MTT and BrdU assays, two-way ANOVA with Dunnett's multiple comparisons test was used. For association of microRNA expression with clinical outcome, microRNA expression was normalized to the mean value of controls (lower expression group) and visualized by box plot. Statistical testing for observed differences was done by two-sided Mann–Whitney *U*-test for related samples with SPSS software version 22 (IBM SPSS Inc., Chicago IL) with the significance level set to *P* = 0.05. The mean value of the lower group in each figure was set to 1.

For *in vivo* experiments, sample size was calculated with GPower Software Version 3.1.7. For testing the hypothesis that tumor size with combination treatment is smaller than with monotherapy by one-sided Student's *t*-test with α = 0.05, β = 0.80, and effect size = 2, necessary sample sizes in each group were calculated as *n* = 4. To compensate for subsequent data loss due to mortality as a cause of treatment, *n* = 5 per group was chosen. For *in vivo* analysis of pre-treated tumor cells, *n* = 4 was chosen per group due to decreased mortality risk. No animals or samples were excluded from any analysis. Animals were randomly assigned to groups for *in vivo* studies. Group allocation and outcome assessment were not done in a blinded manner, including for animal studies. In each group of data, estimated variation was taken into account and is indicated in each figure as SEM. For all graphs, *P = 0.01–0.05, **P = 0.001–0.01, and ***P < 0.001.

### Determination of EC50, EC80, and drug combinations with calculation of the CI

Effective concentrations (EC) were determined as drug doses with half maximal (EC50) or 80% (EC80) of the maximal effect between the upper plateau (no drug) and lower plateau (maximal effect of the drug). For drug combinations, the highest drug dose used was below the EC50 of the drug alone in the respective cell line. The effect of a drug combination was determined according to Breitinger (Berenbaum, 1978; Breitinger, 2012). The combination index (CI) was calculated with a fixed dose for the first drug (here the chemotherapeutic) and the shift in the EC50 for the second drug (here mocetinostat, SAHA did not reached EC50 levels in the applied doses), by applying the following equation: CI = concentration of drug 1 in combination: EC50 drug 1 alone + calculated EC50 of drug 2 in combination: calculated EC50 of drug 2 alone (CI < 1 = synergistic; 1 = no effect; > 1 = antagonistic). MTT assays for drug combinations were run as described above.

### DNA oligonucleotides

**Table:** 

**qPCR**		
β-actin	GCCCTGAGGCACTCTTCCA	TTGCGGATGTCCACGTCA
E-cadherin	GTCCTGGGCAGAGTGAATTT	GACCAAGAAATGGATCTGTGG
Vimentin	CGAGGAGAGCAGGATTTCTC	GGTATCAACCAGAGGGAGTGA
ZEB1	AAGAATTCACAGTGGAGAGAAGCCA	CGTTTCTTGCAGTTTGGGCATT
**Bisulfite sequencing**		
E-cadherin promoter	TTTAGTAATTTTAGGTTAGAGGGTTAT	AAACTCACAAATACTTTACAATTCC
E-cadherin exon 2	GTTTTGTTATTTTGGTTTTGA	AAAACTTACCCATTACAACC
miR-200c, 141	AAGGTTATTAGGGGAGAGGTTT	CTTCAAACCCAAAATCCCTA
miR-203	GAATTCGGGAGGTTAGGTG	ACCCCCTACCCTACTACAACC
**ChIP**		
E-cadherin exon 2	GCCGAGAGCTACACGTTCA	CCAACCCCTCCCTACTCC
miR-200c,141	AGGGCTCACCAGGAAGTGT	AGATCCCTGGCTCCCATC
miR-200b,a,429	CAGCTCGGGCAGCCGTGG	GTCGCTGCGTGCAGGGCTC
miR-203	CGTCTAAGGCGTCCGGTA	GAGCTGCGGAGAGAGGAG

## Supporting Information

Source Data for Supplementary Figure S3

Review Process File
