# Supplementary material for: ZEB1-associated drug resistance in cancer cells is reversed by the class I HDAC inhibitor mocetinostat
Source: EMBO Mol Med. 2015 Apr 14;7(6):831–47. doi: 10.15252/emmm.201404396 (PMC4459821; doi:10.15252/emmm.201404396)
Supplement: Supplementary file 1 [file emmm0007-0831-sd1.pdf]

Supplementary Information

ZEB1-associated drug resistance in cancer cells is reversed by the class I HDAC-inhibitor mocetinostat

Simone Meidhof[*,1,2,3] , Simone Brabletz[*,4] , Waltraut Lehmann[1,3], Bogdan-Tiberius Preca[1,3], Kerstin Mock[1,3], Manuel Ruh[4], Julia Schüler[5], Maria Berthold[1], Anika Weber[1], Ulrike Burk[1], Michael Lübbert[6,10], Martin Puhr[7], Zoran Culig[7], Ulrich Wellner[8], Tobias Keck[8], Peter Bronsert[9], Simon Küsters[1], Ulrich T. Hopt[1], Marc P. Stemmler[4], Thomas Brabletz[4,10]

Table of Contents

Supplementary Figure Legends
Supplementary Figures S1 – S4
Supplementary Tables S1 – S4

Supplementary Figure Legends

Figure S1. miR-203 restores drug sensitivity.

(**A,B**) Stable overexpression of miR-200c and miR-203 in MiaPaCa (A) induces sensitivity to gemcitabine treatment (50nM, 72hrs). In MDA-MB-231 (B), only miR-203 significantly increases sensitivity to paclitaxel at high doses as measured by MTT assay. Overexpression of miR-203 sensitizes MiaPaCa to gemcitabine-triggered (50nM, 48hrs) and MDA to paclitaxel-triggered (5nM, 48hrs) apoptosis as evaluated by cleaved caspase-3 detection in western blot und immunofluorescence. Scale bar 20 µm. n=3, mean ± SEM, Dunnett's multiple comparisons test (p-values in the graphs are: *p=0.01-0.05, **p= 0.001-0.01, ***p<0.001, ****p<0.0001; for exact p-values see Table S4).

(**C**) BrdU incorporation shows enhanced proliferation of miR-203 overexpressing cells. In contrast, miR-203 strongly reduced proliferation in Panc1 and weakly in hPaca1 if combined with gemcitabine treatment (72hrs). n=3, mean ± SEM, Dunnett's multiple comparisons test.

Figure S2. Characterisation of patient derived pancreatic cancer cells and effect of epigenetic drugs on microRNA expression.

(**A**) Immunofluorescence and qRT-PCR showing that the differentiated cell line hPaca2 has an epithelial phenotype, similar to BxPC3, whereas the undifferentiated cell line hPaca1 shows an EMT-phenotype, resembling the cell line Panc1. n=3, mean ± SEM. Scale bar 20 µm. (**B**) The undifferentiated cell line hPaca1, like Panc1, has a CD24+/CD44+ cancer stem cell population. In the differentiated line hPaca2, like in BxPC3, no such population was observed. Dotted lines were added to provide spatial orientation. (**C**) hPaca1 and hPaca2 show similar, but slightly different susceptibilities to gemcitabine and mocetinostat in MTT assays. n=3, mean ± SEM. (**D**) Relative expression levels of indicated genes in Panc1 for all indicated drugs tested. (**E**) Relative expression levels (left panel) in hPaca1 for all drugs and magnification for the effects of the HDACis (right panel). (**F**) Relative expression levels in MDA-MB-231 for treatment with HDACis. (D-F) n= 3, mean ± SEM, unpaired Student's t- test, for detailled analyses of significance see Table S1.

Figure S3. Mocetinostat reduces ZEB1 expression in undifferentiated cancer cells.

(**A**) Western blot and immunofluorescence showing downregulation of ZEB1 and upregulation of acetylated histone 3 and E-cadherin after mocetinostat treatment (1µM, 48hrs) in the undifferentiated cancer cell lines MDA-MB-231 and hPaca1. No change in ZEB1 and E-cadherin expression is seen in the more differentiated patient-derived line hPaca2. Scale bar 20 µm. (**B**) Chromatin immunoprecipitation analysis shows mocetinostat-induced (1µM, 48hrs)

enrichment of the active histone marks H3K4me3, H3ac and H3K9ac at ZEB1 target gene loci in MDA-MB-231. n= 3, mean ± SEM, unpaired Student's t- test. Indicated are only significant differences with *p=0.01-0.05, **p= 0.001-0.01, ***p<0.001. (**C**) Cancer stem cell sphere assay showing reduced sphere-forming capacity of hPaca1 when pre-treated with mocetinostat for 48hrs. n=3, mean ± SEM. ***p<0.001, Mann-Whitney-U test. (**D**) MTT assay showing an increase of relative MTT-activity in Panc1 cells after treatment with antagomirs against miR-203 and all miR-200 family members (a-mix) compared to controls and antagomir against miR-203 alone. This effect was seen in combination of mocetinostat and gemcitabine (Mo plus G) treated cells, but also in cells treated with gemcitabine alone. n=3, mean ± SEM, Dunnett's multiple comparisons test.

Figure S4. Mocetinostat sensitizes to gemcitabine *in vivo*.

(**A**) Relative tumor volume (RTV) in NMRI nu/nu mice with hPaca1 tumors. 18 days after implantation, mice were randomized according to tumor volume. Treatment (day 0) with mocetinostat (60 or 90 mg/kg/day) and gemcitabine (120 mg/kg/day; once a week) was implemented as depicted in the scheme. Shown are the group medians of the RTVs over time (left) and the individual RTVs on day 10 (right). n=5 for each treatment group. *p=0.01-0.05, **p= 0.001-0.01, nonparametric Mann-Whitney-U test. (**B**) Immunohistochemistry of serial sections showing reduced ZEB1 and increased E-cadherin in hPaca1-derived tumors of mice treated with mocetinostat. Scale bar 50 µm, inserts for higher magnifications 20 µm. (**C**) *In situ* hybridisation (isH) for miR-203 and control probe shows gain of miR-203 and associated loss of ZEB1 detected by immunohistochemistry in serial sections of mocetinostat treated hPaca1-derived tumors. Scale bar 50 µm, inserts for higher magnifications 15 µm. (**D**) Relative tumor volume (RTV) in NMRI nu/nu mice with hPaca2-derived tumors. 10 days after implantation, mice were randomized according to tumor volume and treatment with mocetinostat (60 mg/kg/day) and gemcitabine (25 mg/kg/day; twice a week) was implemented as depicted in the scheme. Shown are the group medians of the RTVs over time (left) and the individual RTVs on day 32 (right). n=5 for each treatment group. *p=0.01-0.05, nonparametric Mann-Whitney-U test. (**E**) Immunohistochemistry of serial sections showing no change in ZEB1 and E-cadherin expression in hPaca2-derived tumors of mice treated with mocetinostat. Scale bar 50 µm, inserts for higher magnifications 20 µm.

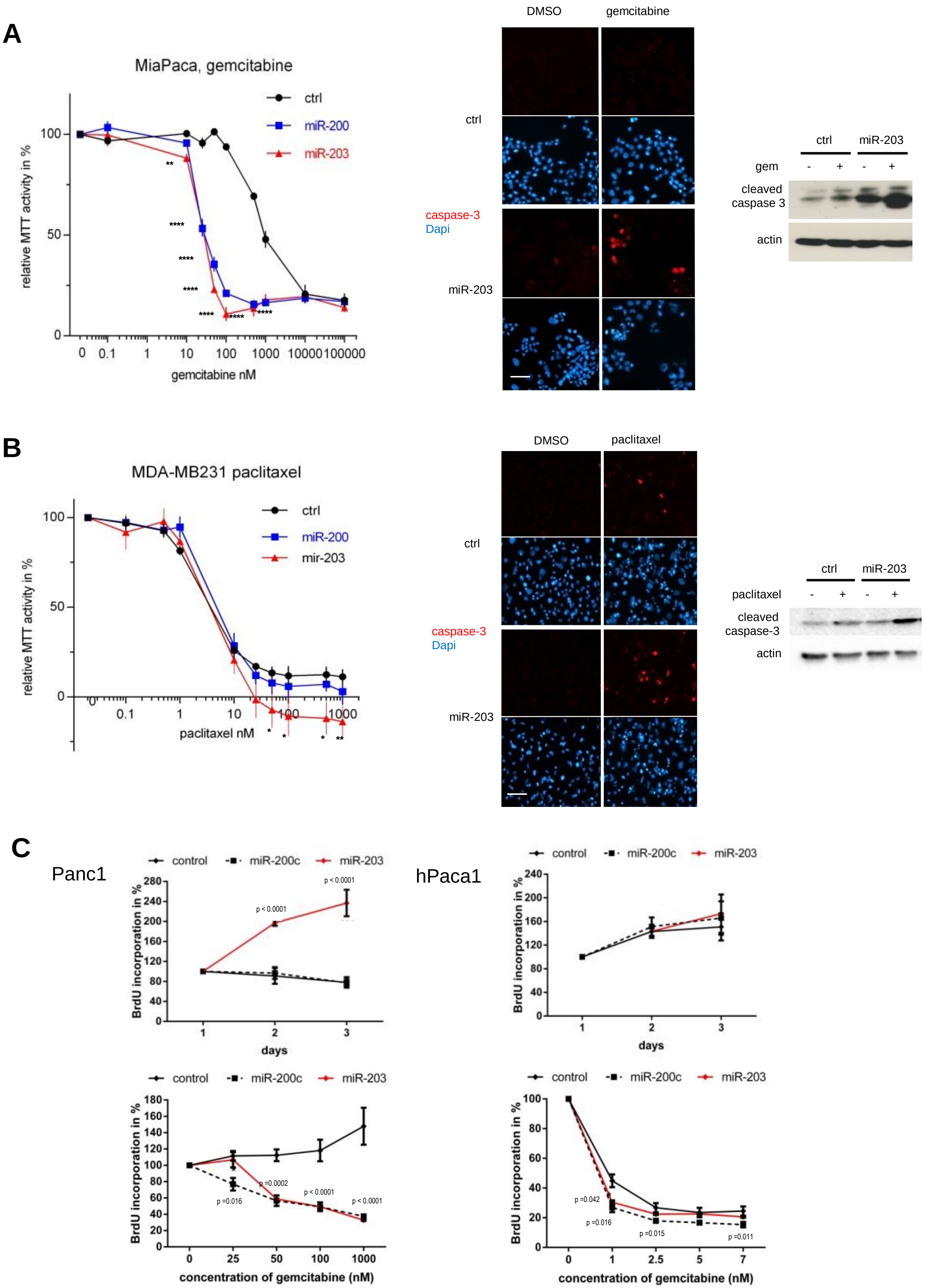

Meidhof et al. Fig. S1

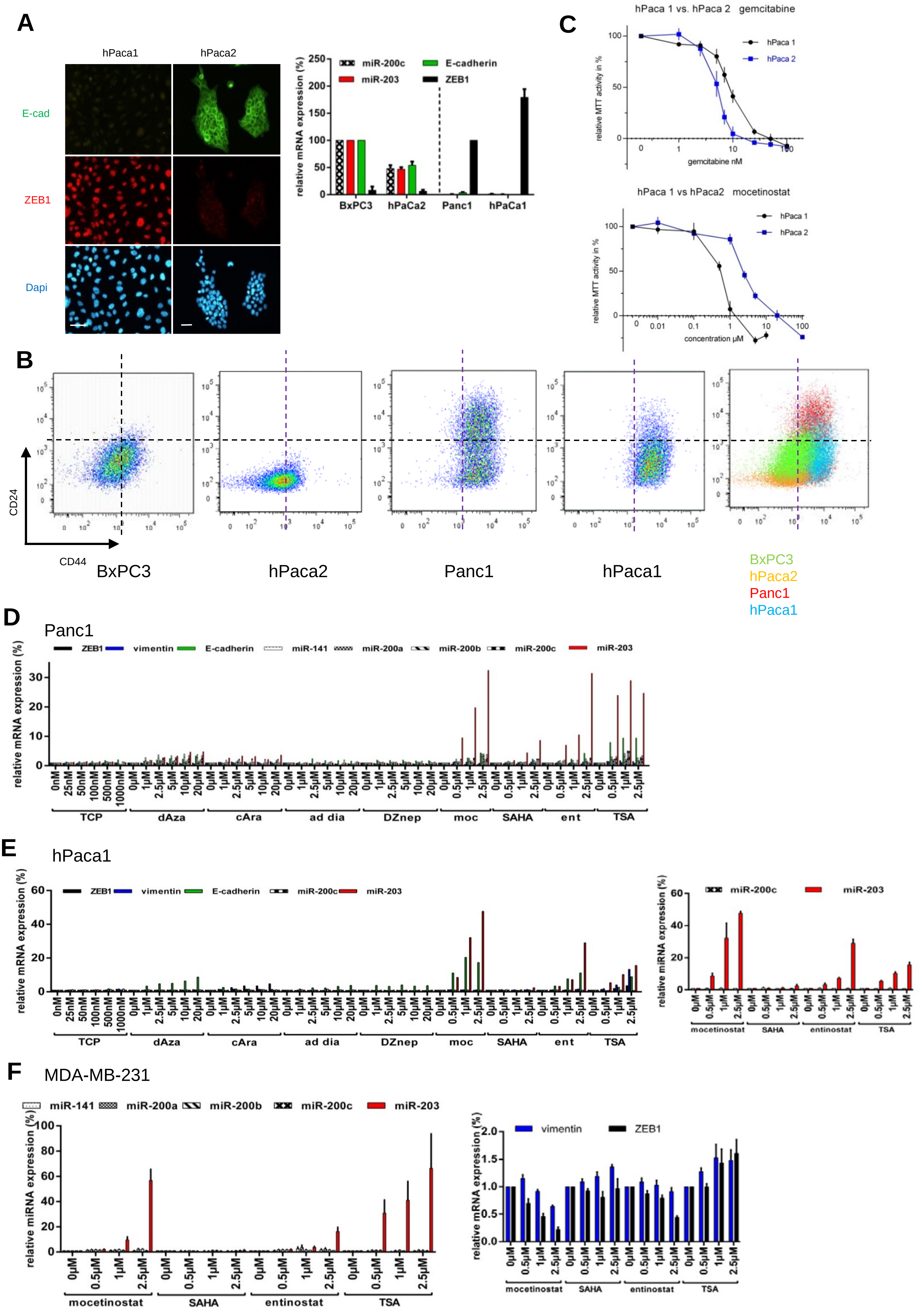

Meidhof et al. Fig. S2

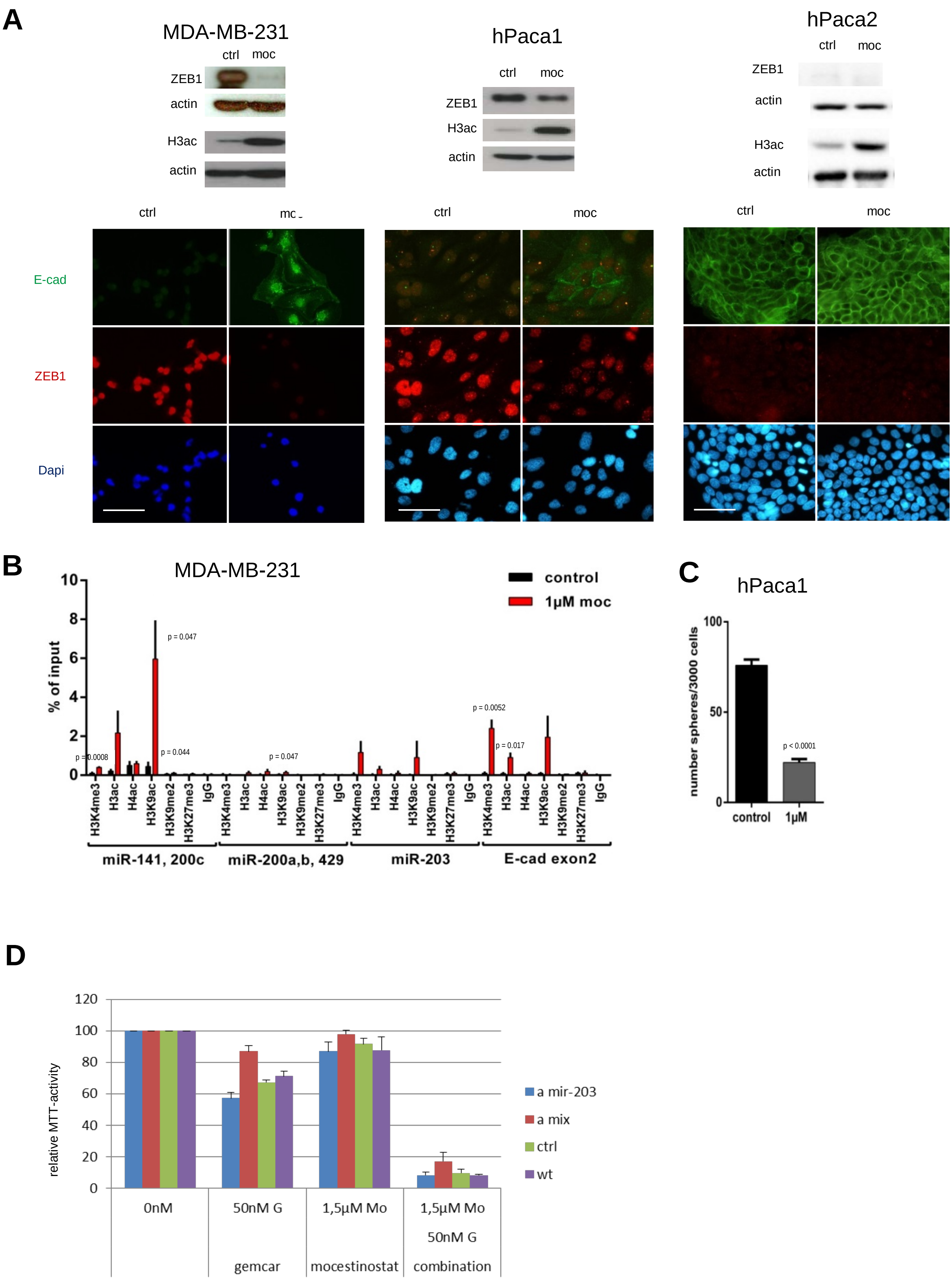

Meidhof et al. Fig. S3

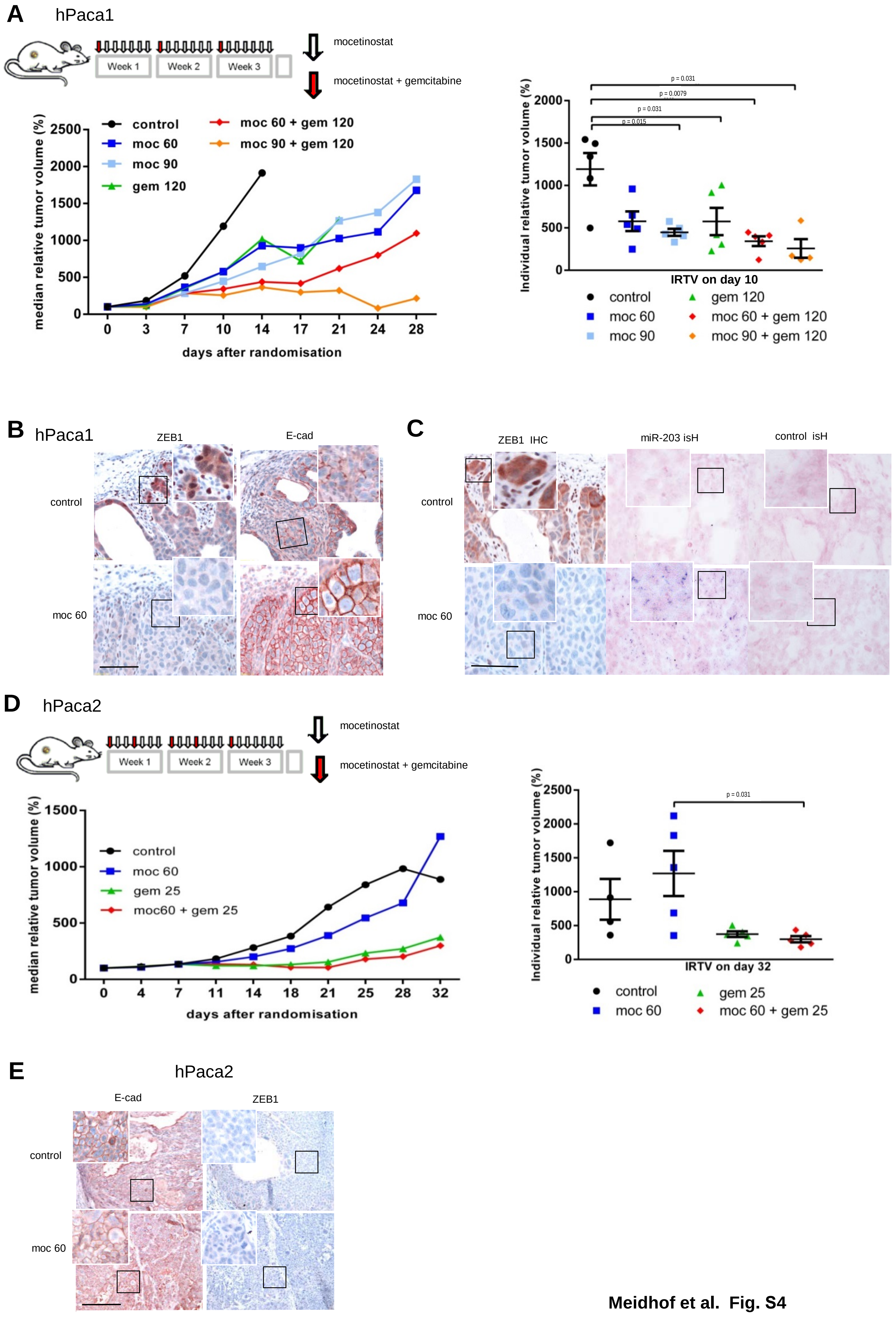

Meidhof et al. Fig. S4

Supplementary Tables

Table S1. Statistics to the drug screens in Fig. 2 A,B and Fig. S2 D-F.

				significance	P value
**Panc1**	Mocetinostat	0µM vs 0.5µM	miR-141	*	0.0101472
**to Fig. 2 A,B and suppl Fig. 2 D**			miR-200a	*	0.00178797
			miR-200b	*	0.0192398
			miR-200c	*	0.000296977
			miR-203	*	0.00177692
		0µM vs 1µM	miR-141		0.0904472
			miR-200a		0.169698
			miR-200b	*	0.0275343
			miR-200c	*	7.23038E-05
			miR-203	*	0.00657138
		0µM vs 2.5µM	miR-141	*	0.00501117
			miR-200a	*	0.00307866
			miR-200b	*	0.000226343
			miR-200c	*	0.000763524
			miR-203	*	0.000886167
	SAHA	0µM vs 0.5µM	miR-141	*	0.00685916
			miR-200a	*	0.00796948
			miR-200b		0.927451
			miR-200c		0.620388
			miR-203		0.313761
		0µM vs 1µM	miR-141	*	0.00398095
			miR-200a	*	0.0312312
			miR-200b		0.408944
			miR-200c		0.408467
			miR-203		0.159719
		0µM vs 2.5µM	miR-141	*	0.00169659
			miR-200a	*	0.0197391
			miR-200b		0.08615
			miR-200c		0.294927
			miR-203		0.0523112
	Entinostat	0µM vs 0.5µM	miR-141		0.0524772
			miR-200a		0.101803
			miR-200b		0.229399
			miR-200c	*	0.000705014
			miR-203	*	0.0309337
		0µM vs 1µM	miR-141		0.0619428
			miR-200a		0.761706
			miR-200b		0.339964
			miR-200c	*	0.00906543
			miR-203	*	0.0311997
		0µM vs 2.5µM	miR-141	*	0.0110309
			miR-200a		0.144303
			miR-200b		0.37936
			miR-200c	*	2.48221E-05

			miR-203	*	0.00183598
	TSA	0µM vs 0.5µM	miR-141	*	0.0242689
			miR-200a		0.0574231
			miR-200b	*	0.0179132
			miR-200c	*	0.00573301
			miR-203	*	0.00588348
		0µM vs 1µM	miR-141		0.296637
			miR-200a		0.232213
			miR-200b		0.237649
			miR-200c	*	0.0125326
			miR-203	*	0.0314868
		0µM vs 2.5µM	miR-141		0.217921
			miR-200a	*	0.0408224
			miR-200b		0.0523826
			miR-200c	*	0.0070874
			miR-203	*	0.00959804
	TCP	0nM vs 25nM	miR-141		0.398226
			miR-200a		0.813579
			miR-200b	*	2.39516E-05
			miR-200c	*	0.00169085
			miR-203	*	0.0214136
		0nM vs 50nM	miR-141		0.910641
			miR-200a		0.865217
			miR-200b		0.0889865
			miR-200c		0.316396
			miR-203		0.243373
		0nM vs 100nM	miR-141		0.941126
			miR-200a		0.791931
			miR-200b		0.771651
			miR-200c		0.891784
			miR-203	*	0.0310799
		0nM vs 500nM	miR-141		0.509422
			miR-200a		0.722912
			miR-200b	*	0.000100154
			miR-200c		0.319105
			miR-203	*	0.00240042
		0nM vs 1000nM	miR-141	*	0.00148633
			miR-200a	*	0.00535623
			miR-200b		0.10761
			miR-200c		0.157046
			miR-203		0.4663
	dAza	0µM vs 1µM	miR-141	*	0.00337048
			miR-200a	*	0.0384273
			miR-200b	*	0.000772014
			miR-200c	*	0.0245871
			miR-203		0.150975
		0µM vs 2.5µM	miR-141	*	0.0423177
			miR-200a		0.149949
			miR-200b		0.100686
			miR-200c	*	0.0185476
			miR-203	*	0.0367578
		0µM vs 5µM	miR-141	*	0.0189602
			miR-200a	*	0.0383052

			miR-200b	*	0.0396054
			miR-200c	*	0.0159626
			miR-203	*	0.00652072
		0µM vs 10µM	miR-141	*	0.00271638
			miR-200a		0.0705231
			miR-200b		0.102657
			miR-200c	*	0.00492039
			miR-203	*	0.0132818
		0µM vs 20µM	miR-141		0.05638
			miR-200a		0.11147
			miR-200b		0.0755656
			miR-200c	*	0.00146715
			miR-203	*	5.9117E-05
	cAra	0µM vs 1µM	miR-141		0.756254
			miR-200a		0.874297
			miR-200b	*	0.0435609
			miR-200c		0.118404
			miR-203	*	0.0373731
		0µM vs 2.5µM	miR-141		0.44783
			miR-200a		0.334982
			miR-200b	*	0.000639399
			miR-200c		0.109202
			miR-203		0.0735663
		0µM vs 5µM	miR-141		0.710916
			miR-200a		0.636781
			miR-200b	*	0.000308005
			miR-200c	*	0.0292408
			miR-203	*	0.00958409
		0µM vs 10µM	miR-141		0.415608
			miR-200a		0.957821
			miR-200b	*	1.63546E-05
			miR-200c		0.17101
			miR-203		0.318972
		0µM vs 20µM	miR-141		0.880276
			miR-200a		0.235791
			miR-200b	*	0.0292875
			miR-200c		0.231039
			miR-203		0.0763708
	ad dia	0µM vs 1µM	miR-141		0.11692
			miR-200a		0.405597
			miR-200b		0.97796
			miR-200c		0.681376
			miR-203		0.892451
		0µM vs 2.5µM	miR-141	*	0.0100483
			miR-200a		0.54253
			miR-200b		0.139173
			miR-200c	*	0.0120456
			miR-203		0.960664
		0µM vs 5µM	miR-141		0.14102
			miR-200a	*	0.00490898
			miR-200b		0.426241
			miR-200c	*	0.0335828
			miR-203		0.228684

		0µM vs 10µM	miR-141	*	4.80373E-05
			miR-200a		0.109981
			miR-200b		0.540626
			miR-200c		0.162942
			miR-203		0.449171
		0µM vs 20µM	miR-141		0.288932
			miR-200a		0.385234
			miR-200b		0.161664
			miR-200c	*	0.0114124
			miR-203		0.700658
	Dznep	0µM vs 1µM	miR-141		0.491021
			miR-200a		0.191658
			miR-200b		0.275624
			miR-200c		0.241271
			miR-203		0.671703
		0µM vs 2.5µM	miR-141	*	0.00522901
			miR-200a	*	1.7797E-08
			miR-200b		0.583957
			miR-200c		0.190416
			miR-203	*	0.0121261
		0µM vs 5µM	miR-141		0.25594
			miR-200a	*	0.000724519
			miR-200b		0.670705
			miR-200c		0.451233
			miR-203		0.840981
		0µM vs 10µM	miR-141		
			miR-200a		
			miR-200b		
			miR-200c		0.204622
			miR-203		0.192553
		0µM vs 20µM	miR-141		0.0536515
			miR-200a		0.95336
			miR-200b	*	0.0172065
			miR-200c	*	0.0171137
			miR-203		0.0524531
	Mocetinostat	0µM vs 0.5µM	E-cadherin	*	0.00695611
			ZEB1	*	4.40891E-05
		0µM vs 1µM	E-cadherin	*	0.000297856
			ZEB1	*	3.72811E-05
		0µM vs 2.5µM	E-cadherin	*	1.73154E-05
			ZEB1	*	2.46651E-05
	SAHA	0µM vs 0.5µM	E-cadherin		0.126415
			ZEB1	*	0.0272728
		0µM vs 1µM	E-cadherin		0.353085
			ZEB1	*	0.000117849
		0µM vs 2.5µM	E-cadherin		0.277507
			ZEB1	*	0.00126674
	Entinostat	0µM vs 0.5µM	E-cadherin		0.0732973
			ZEB1	*	0.00766255
		0µM vs 1µM	E-cadherin	*	0.00528906
			ZEB1	*	0.00384373
		0µM vs 2.5µM	E-cadherin	*	0.00427483
			ZEB1	*	6.52404E-06

	TSA	0µM vs 0.5µM	E-cadherin	*	0.00312549
			ZEB1	*	0.0132146
		0µM vs 1µM	E-cadherin	*	0.00337924
			ZEB1		0.0811354
		0µM vs 2.5µM	E-cadherin	*	0.00475107
			ZEB1		0.103332
	TCP	0nM vs 25nM	E-cadherin		0.340314
			ZEB1		0.0910166
		0nM vs 50nM	E-cadherin		0.215017
			ZEB1	*	0.00191769
		0nM vs 100nM	E-cadherin		0.215452
			ZEB1		0.290918
		0nM vs 500nM	E-cadherin		0.255421
			ZEB1		0.158238
		0nM vs 1000nM	E-cadherin		0.00372432
			ZEB1		0.704464
	dAza	0µM vs 1µM	E-cadherin		0.16989
			ZEB1		0.983178
		0µM vs 2.5µM	E-cadherin	*	0.000609584
			ZEB1		0.88983
		0µM vs 5µM	E-cadherin	*	0.00305659
			ZEB1		0.729738
		0µM vs 10µM	E-cadherin	*	0.0478358
			ZEB1		0.774152
		0µM vs 20µM	E-cadherin	*	0.00019573
			ZEB1		0.858412
	cAra	0µM vs 1µM	E-cadherin	*	0.0119414
			ZEB1		0.348747
		0µM vs 2.5µM	E-cadherin		0.582059
			ZEB1		0.0903216
		0µM vs 5µM	E-cadherin		0.208189
			ZEB1		0.290854
		0µM vs 10µM	E-cadherin		0.291457
			ZEB1		0.462611
		0µM vs 20µM	E-cadherin		0.0593593
			ZEB1	*	0.00429863
	ad dia	0µM vs 1µM	E-cadherin		0.125701
			ZEB1		0.574246
		0µM vs 2.5µM	E-cadherin		0.272686
			ZEB1		0.284332
		0µM vs 5µM	E-cadherin		0.255639
			ZEB1	*	0.0373762
		0µM vs 10µM	E-cadherin		0.279453
			ZEB1	*	0.0320593
		0µM vs 20µM	E-cadherin		0.403449
			ZEB1		0.128064
	Dznep	0µM vs 1µM	E-cadherin		0.122818
			ZEB1	*	0.0442705
		0µM vs 2.5µM	E-cadherin	*	0.000797316
			ZEB1	*	0.0201122
		0µM vs 5µM	E-cadherin	*	0.000417205
			ZEB1	*	0.000365268
		0µM vs 10µM	E-cadherin		0.181355

			ZEB1		0.0678065
		0µM vs 20µM	E-cadherin	*	4.83467E-05
			ZEB1		0.175484
**hPaca1**	TCP	0nM vs 25nM	ZEB1	*	0.0369032
**to suppl Fig. 2 E**			E-cad		0.383551
			miR-200c		0.450364
		0nM vs 50nM	ZEB1		0.673638
			E-cad		0.140315
			miR-200c		0.488787
		0nM vs 100nM	ZEB1		0.804466
			E-cad		0.295539
			miR-200c		0.45576
		0nM vs 500nM	ZEB1		0.476616
			E-cad		0.273778
			miR-200c		0.343517
		0nM vs 1000nM	ZEB1	*	0.000866533
			E-cad		0.206058
			miR-200c		0.260034
	dAza	0µM vs 1µM	ZEB1	*	0.00831137
			E-cad		0.0578366
			miR-200c	*	0.0339567
		0µM vs 2.5µM	ZEB1		0.58519
			E-cad	*	0.0301748
			miR-200c	*	0.000174277
		0µM vs 5µM	ZEB1		0.347428
			E-cad	*	0.00920103
			miR-200c		0.251121
		0µM vs 10µM	ZEB1		0.0993513
			E-cad	*	0.0071647
			miR-200c		0.115504
		0µM vs 20µM	ZEB1		0.0689343
			E-cad	*	0.00417792
			miR-200c	*	0.0437705
	cAra	0µM vs 1µM	ZEB1	*	0.00158253
			E-cad	*	0.00257191
			miR-200c	*	0.0206921
		0µM vs 2.5µM	ZEB1	*	0.0313589
			E-cad		0.0959373
			miR-200c		0.152889
		0µM vs 5µM	ZEB1	*	0.0383492
			E-cad		0.263461
			miR-200c		0.395634
		0µM vs 10µM	ZEB1	*	0.0162068
			E-cad		0.120288
			miR-200c		0.167271
		0µM vs 20µM	ZEB1		0.057495
			E-cad		0.826959
			miR-200c		0.253215
	Ad dia	0µM vs 1µM	ZEB1		0.435266
			E-cad	*	0.000104974
			miR-200c		0.435716
		0µM vs 2.5µM	ZEB1		0.368703

			E-cad		0.1254
			miR-200c		0.622071
		0µM vs 5µM	ZEB1	*	0.00226961
			E-cad	*	0.0293654
			miR-200c		0.392492
		0µM vs 10µM	ZEB1	*	0.0290271
			E-cad		0.050325
			miR-200c		0.336394
		0µM vs 20µM	ZEB1		0.139624
			E-cad	*	0.0165247
			miR-200c		0.46031
	Dznep	0µM vs 1µM	ZEB1		0.380529
			E-cad	*	0.00193867
			miR-200c	*	0.00384111
		0µM vs 2.5µM	ZEB1		0.218106
			E-cad	*	0.018505
			miR-200c		0.272617
		0µM vs 5µM	ZEB1		0.0522933
			E-cad	*	0.0121876
			miR-200c		0.0635157
		0µM vs 10µM	ZEB1	*	0.00371306
			E-cad	*	0.000500418
			miR-200c		0.0584035
		0µM vs 20µM	ZEB1		0.0953649
			E-cad	*	0.0140657
			miR-200c		0.360375
	Mocetinostat	0µM vs 0.5µM	ZEB1	*	0.0484159
			E-cad	*	0.00908883
			miR-200c		0.937541
			miR-203		0.0527073
		0µM vs 1µM	ZEB1		0.322688
			E-cad	*	0.03249
			miR-200c		0.384994
			miR-203		0.0820926
		0µM vs 2.5µM	ZEB1		0.339096
			E-cad	*	0.000241696
			miR-200c		0.805699
			miR-203	*	0.000603601
	SAHA	0µM vs 0.5µM	ZEB1		0.542816
			E-cad		0.802273
			miR-200c		0.40171
			miR-203		0.83245
		0µM vs 1µM	ZEB1		0.7626
			E-cad		0.71762
			miR-200c	*	0.000401118
			miR-203		0.381133
		0µM vs 2.5µM	ZEB1		0.464855
			E-cad		0.278949
			miR-200c		0.238344
			miR-203		0.22883
	Entinostat	0µM vs 0.5µM	ZEB1		0.136156
			E-cad		0.0614782
			miR-200c		0.306586

			miR-203		0.167243
		0µM vs 1µM	ZEB1		0.200884
			E-cad	*	0.000134667
			miR-200c		0.926642
			miR-203	*	0.00678079
		0µM vs 2.5µM	ZEB1		0.083922
			E-cad	*	6.68727E-05
			miR-200c		0.582187
			miR-203	*	0.00821309
	TSA	0µM vs 0.5µM	ZEB1		0.136156
			E-cad		0.0614782
			miR-200c		0.306586
			miR-203		0.167243
		0µM vs 1µM	ZEB1		0.0739032
			E-cad		0.344408
			miR-200c		0.99663
			miR-203	*	0.0074817
		0µM vs 2.5µM	ZEB1	*	0.00126273
			E-cad	*	0.00451344
			miR-200c	*	0.00377864
			miR-203	*	0.012534
**MDA-MB-231**	Mocetinostat	0µM vs 0.5µM	ZEB1	*	0.02774
**to suppl Fig. 2 F**			E-cad	*	0.00009
			miR-141	*	0.00009
			miR-200a	*	0.00009
			miR-200b	*	0.00009
			miR-200c	*	0.00009
			miR-203	*	0.00009
		0µM vs 1µM	ZEB1	*	0.00009
			E-cad	*	0.00009
			miR-141		0.38062
			miR-200a		0.05284
			miR-200b		0.05805
			miR-200c		0.17535
			miR-203	*	0.02483
		0µM vs 2.5µM	ZEB1	*	0.00004
			E-cad	*	0.00010
			miR-141		0.90282
			miR-200a		0.07819
			miR-200b	*	0.00668
			miR-200c		0.43628
			miR-203	*	0.00338
	SAHA	0µM vs 0.5µM	ZEB1		0.13936
			E-cad	*	0.03003
			miR-141		0.57542
			miR-200a		0.28114
			miR-200b	*	0.04456
			miR-200c		0.82448
			miR-203	*	0.02154
		0µM vs 1µM	ZEB1		0.14086
			E-cad		0.60005

			miR-141		0.40008
			miR-200a		0.05553
			miR-200b		0.09950
			miR-200c		0.16721
			miR-203	*	0.02503
		0µM vs 2.5µM	ZEB1		0.84938
			E-cad		0.11953
			miR-141		0.21660
			miR-200a		0.07093
			miR-200b		0.81734
			miR-200c	*	0.04061
			miR-203		0.57623
	Entinostat	0µM vs 0.5µM	ZEB1		0.07714
			E-cad	*	0.02583
			miR-141		0.99209
			miR-200a		0.12118
			miR-200b		0.12717
			miR-200c	*	0.01300
			miR-203		0.05093
		0µM vs 1µM	ZEB1	*	0.01838
			E-cad	*	0.00178
			miR-141		0.37090
			miR-200a		0.29809
			miR-200b		0.12913
			miR-200c	*	0.00079
			miR-203	*	0.03295
		0µM vs 2.5µM	ZEB1	*	0.00004
			E-cad	*	0.00232
			miR-141		0.39099
			miR-200a		0.18380
			miR-200b		0.14264
			miR-200c	*	0.02478
			miR-203	*	0.01222
	TSA	0µM vs 0.5µM	ZEB1		0.93799
			E-cad	*	0.01322
			miR-141		0.47614
			miR-200a		0.61435
			miR-200b	*	0.02581
			miR-200c	*	0.04880
			miR-203	*	0.04788
		0µM vs 1µM	ZEB1		0.17481
			E-cad	*	0.00390
			miR-141	*	0.02319
			miR-200a		0.83872
			miR-200b	*	0.01996
			miR-200c		0.76332
			miR-203		0.05465
		0µM vs 2.5µM	ZEB1		0.06895
			E-cad	*	0.00217
			miR-141		0.23513
			miR-200a		0.32887
			miR-200b		0.63997
			miR-200c		0.62792

			miR-203		0.07623

Table S2. Clinical data of investigated pancreatic adenocarcinomas vs. adjacent normal pancreatic epithelium.

case no.	sex	localisation	TNM-status	grading	R-classif.	recurrence	ZEB1 staining
1	m	pancreas head	pT4pN0Mx	2	0	-	-
2	m	pancreas head	pT3pN1pMx	2	0	-	-
3	f	pancreas head	pT3pN0pMx	2	0	-	-
4	f	pancreas head	pT3pN1pMx	2	0	-	-
5	m	pancreas head	pT3pN1pMx	3	0	local	+
6	f	pancreas head	pT3pN1pMx	3	0	local, met	+

Table S3. Clinical data of investigated pancreatic adenocarcinomas with early vs. no recurrence.

case no.	disease recurrence	sex	localisation	TNM-status	grading	R-classif.	recurrence	ZEB1 staining
1	no > 2 years	m	pancreas head	pT4pN0Mx	2	0	-	-
2	no > 2 years	f	pancreas head	pT3pN1pMx	2	0	-	-
3	no > 2 years	m	pancreas head	pT3pN1Mx	2	0	-	-
4	no > 2 years	m	pancreas head	pT3pN1pMx	2	0	-	-
5	no > 2 years	f	pancreas head	pT3pN0pMx	2	0	-	-
6	no > 2 years	f	pancreas corpus	pT3pN1pMx	2	0	-	-
7	no > 2 years	f	pancreas head	pT3pN1pMx	2	0	-	-
8	no > 2 years	m	pancreas head	pT3pN0pMx	2	0	-	-
9	no > 2 years	f	pancreas head	pT3pN1pMx	2	0	-	-
10	no > 2 years	m	pancreas corpus	pT3pN0pMx	2	0	-	-
11	yes <6 months	m	pancreas head	pT3pN1pM0	2	0	local	+
12	yes <6 months	m	pancreas head	pT3 pN1Mx	2	0	met	-
13	yes <6 months	f	pancreas head	pT3pN1Mx	2	0	met	+
14	yes <6 months	m	pancreas head	pT3pN1Mx	2	0	local	+
15	yes <6 months	m	pancreas head	pT3pN1pMx	3	0	local	+
16	yes <6 months	f	pancreas head	pT3pN1pMx	3	0	local	-
17	yes <6 months	m	pancreas head	pT3pN1pMx	2	0	local	-
18	yes <6 months	m	pancreas head	pT3pN1pMx	3	0	local	-
19	yes <6 months	f	pancreas head	pT3pN1pMx	3	0	local, met	+
20	yes <6 months	f	pancreas head	pT3pN1pMx	3	0	local, met	+
21	yes <6 months	m	pancreas corpus	pT3pN1pMx	2	0	local	0

Table S4: Exact p-values for multiple comparisons statistical analyses of MTT-assays
(Dunnett' multiple comparisons test, only for graphs reaching statistical significance).

Fig. 1B, Panc1		p-value
Row 1		
ctrl vs. miR-200	ns	> 0,9999
ctrl vs. miR-203	ns	> 0,9999
Row 2		
ctrl vs. miR-200	ns	0,9728
ctrl vs. miR-203	ns	0,8369
Row 3		
ctrl vs. miR-200	ns	0,1913
ctrl vs. miR-203	ns	0,9749
Row 4		
ctrl vs. miR-200	*	0,0113
ctrl vs. miR-203	ns	0,3490
Row 5		
ctrl vs. miR-200	ns	0,0689
ctrl vs. miR-203	ns	0,3880
Row 6		
ctrl vs. miR-200	*	0,0147
ctrl vs. miR-203	****	< 0,0001
Row 7		
ctrl vs. miR-200	*	0,0216
ctrl vs. miR-203	****	< 0,0001
Row 8		
ctrl vs. miR-200	*	0,0413
ctrl vs. miR-203	****	< 0,0001
Row 9		
ctrl vs. miR-200	*	0,0166
ctrl vs. miR-203	****	< 0,0001
Row 10		
ctrl vs. miR-200	**	0,0053
ctrl vs. miR-203	****	< 0,0001
Row 11		
ctrl vs. miR-200	*	0,0144
ctrl vs. miR-203	****	< 0,0001
Row 12		
ctrl vs. miR-200	*	0,0191
ctrl vs. miR-203	****	< 0,0001
Row 13		
ctrl vs. miR-200	ns	0,2613
ctrl vs. miR-203	****	< 0,0001

Fig. 1B, hPaca1		
ctrl vs. miR-200	ns	> 0,9999
ctrl vs. miR-203	ns	> 0,9999
Row 2		
ctrl vs. miR-200	ns	0,9372
ctrl vs. miR-203	ns	0,7058
Row 3		
ctrl vs. miR-200	ns	0,5284
ctrl vs. miR-203	ns	0,7673
Row 4		
ctrl vs. miR-200	ns	0,3581
ctrl vs. miR-203	****	< 0,0001
Row 5		
ctrl vs. miR-200	ns	0,2267
ctrl vs. miR-203	****	< 0,0001
Row 6		
ctrl vs. miR-200	ns	0,0538
ctrl vs. miR-203	****	< 0,0001
Row 7		
ctrl vs. miR-200	ns	0,1079
ctrl vs. miR-203	****	< 0,0001
Row 8		
ctrl vs. miR-200	**	0,0042
ctrl vs. miR-203	****	< 0,0001
Row 9		
ctrl vs. miR-200	**	0,0020
ctrl vs. miR-203	***	0,0001

Fig. 1D		
Row 1		
ctrl vs. anta miR-200	ns	> 0,9999

ctrl vs. anta miR-203	ns	> 0,9999
Row 2		
ctrl vs. anta miR-200	ns	0,2825
ctrl vs. anta miR-203	ns	0,4758
Row 3		
ctrl vs. anta miR-200	ns	0,1004
ctrl vs. anta miR-203	ns	0,1301
Row 4		
ctrl vs. anta miR-200	**	0,0013
ctrl vs. anta miR-203	**	0,0013

Fig. 4B left

Row 1		
moc alone vs. 25 nM gemzar +moc	*	0,0265
moc alone vs. 50 nM gemzar +moc	***	0,0004
Row 2		
moc alone vs. 25 nM gemzar +moc	****	< 0,0001
moc alone vs. 50 nM gemzar +moc	****	< 0,0001
Row 3		
moc alone vs. 25 nM gemzar +moc	****	< 0,0001
moc alone vs. 50 nM gemzar +moc	****	< 0,0001

Fig. 4D right

Row 1		
mocetinostat alone vs. mocetinostat + gemzar	ns	0,9976
mocetinostat alone vs. Group C	ns	0,9976
Row 2		
mocetinostat alone vs. mocetinostat + gemzar	*	0,0388
mocetinostat alone vs. Group C	*	0,0388
Row 3		
mocetinostat alone vs. mocetinostat + gemzar	*	0,0168
mocetinostat alone vs. Group C	*	0,0168

Fig. S1A

Row 1		
ctrl vs. miR-200	ns	> 0,9999
ctrl vs. miR-203	ns	> 0,9999
Row 2		
ctrl vs. miR-200	ns	0,1603
ctrl vs. miR-203	ns	0,6808
Row 3		
ctrl vs. miR-200	ns	0,3736
ctrl vs. miR-203	**	0,0050
Row 4		
ctrl vs. miR-200	****	< 0,0001
ctrl vs. miR-203	****	< 0,0001
Row 5		
ctrl vs. miR-200	****	< 0,0001
ctrl vs. miR-203	****	< 0,0001
Row 6		
ctrl vs. miR-200	****	< 0,0001
ctrl vs. miR-203	****	< 0,0001
Row 7		
ctrl vs. miR-200	****	< 0,0001
ctrl vs. miR-203	****	< 0,0001
Row 8		
ctrl vs. miR-200	****	< 0,0001
ctrl vs. miR-203	****	< 0,0001
Row 9		
ctrl vs. miR-200	ns	0,8312
ctrl vs. miR-203	ns	0,9373
Row 10		
ctrl vs. miR-200	ns	0,9858
ctrl vs. miR-203	ns	0,5571

Fig. S1B

Row 1		
ctrl vs. miR-200	ns	> 0,9999
ctrl vs. miR-203	ns	> 0,9999
Row 2		
ctrl vs. miR-200	ns	0,9982
ctrl vs. miR-203	ns	0,7874
Row 3		
ctrl vs. miR-200	ns	0,9988
ctrl vs. miR-203	ns	0,7748
Row 4		
ctrl vs. miR-200	ns	0,2247

ctrl vs. miR-203	ns	0,7683
Row 5		
ctrl vs. miR-200	ns	0,9328
ctrl vs. miR-203	ns	0,7658
Row 6		
ctrl vs. miR-200	ns	0,7802
ctrl vs. miR-203	ns	0,0645
Row 7		
ctrl vs. miR-200	ns	0,7404
ctrl vs. miR-203	*	0,0362
Row 8		
ctrl vs. miR-200	ns	0,7213
ctrl vs. miR-203	*	0,0212
Row 9		
ctrl vs. miR-200	ns	0,7565
ctrl vs. miR-203	*	0,0117
Row 10		
ctrl vs. miR-200	ns	0,5306
ctrl vs. miR-203	**	0,0098
