# Supplementary figures and images for: ZEB1-associated drug resistance in cancer cells is reversed by the class I HDAC inhibitor mocetinostat
Source: EMBO Mol Med. 2015 Apr 14;7(6):831–47. doi: 10.15252/emmm.201404396 (PMC4459821; doi:10.15252/emmm.201404396)

Figure S1A

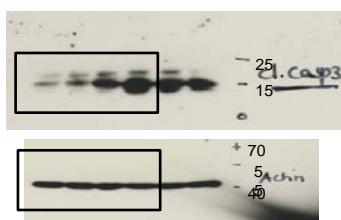

Figure S1B

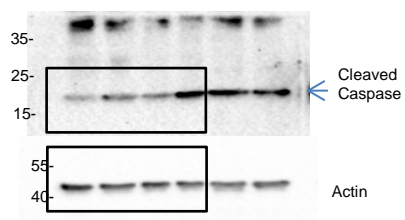

Supplement: Supplementary file 2 [file emmm0007-0831-sd2.pdf]

Figure S3A

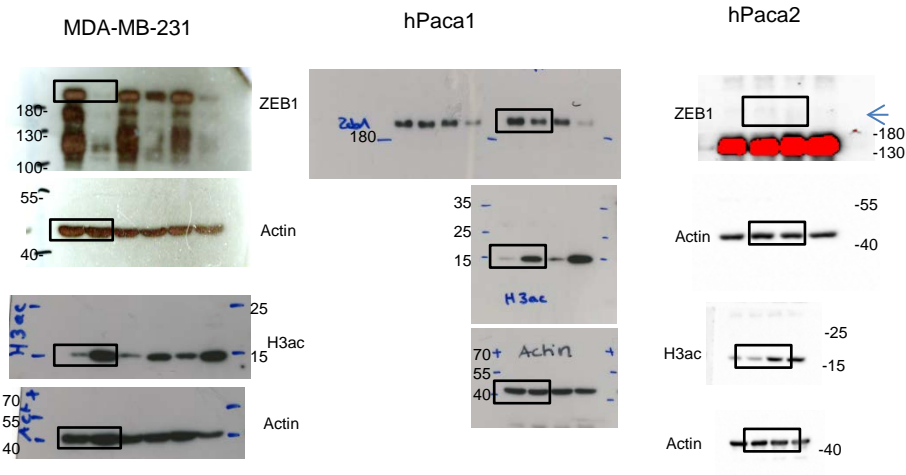

Supplement: Supplementary file 3 [file emmm0007-0831-sd3.pdf]

Figure source data, Fig. 1

Figure 1 A

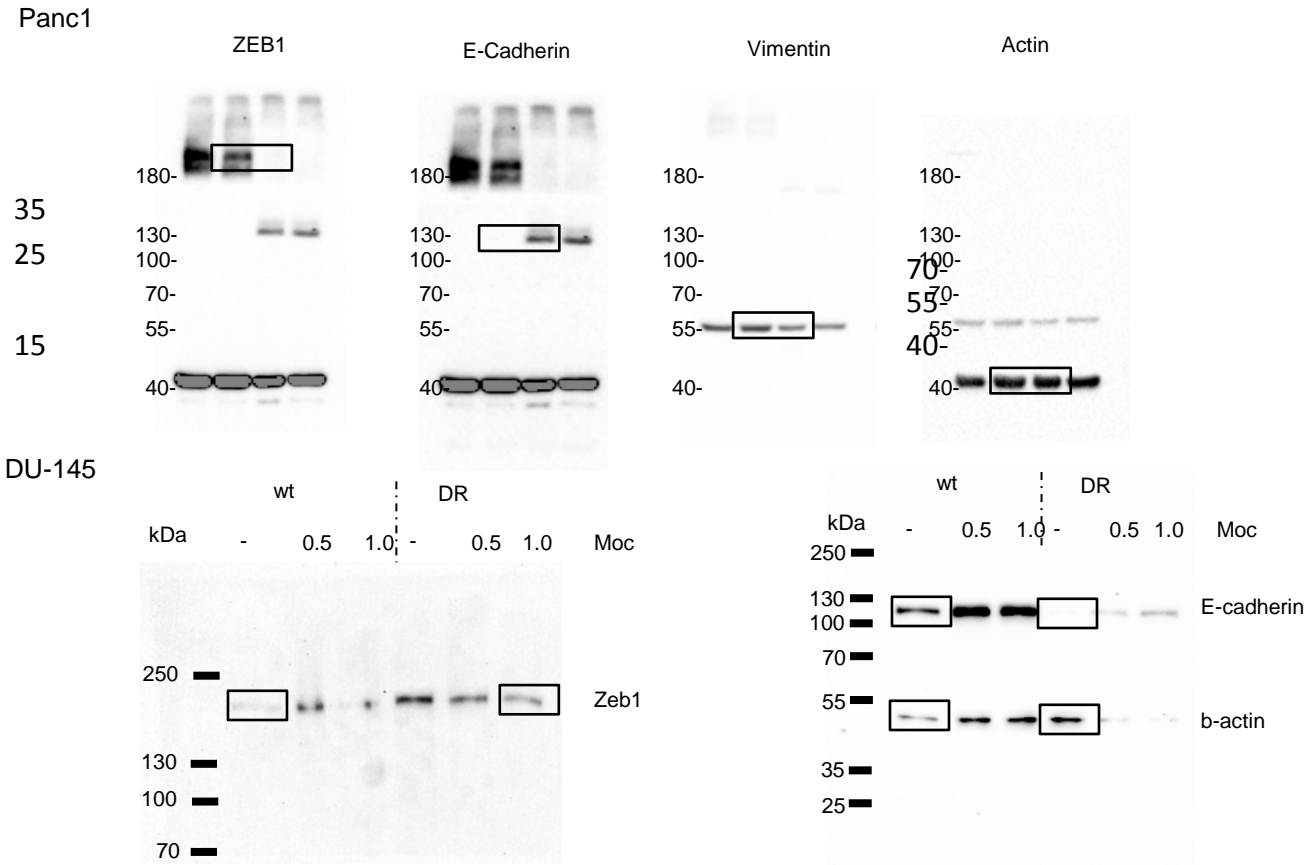

Figure 1 C

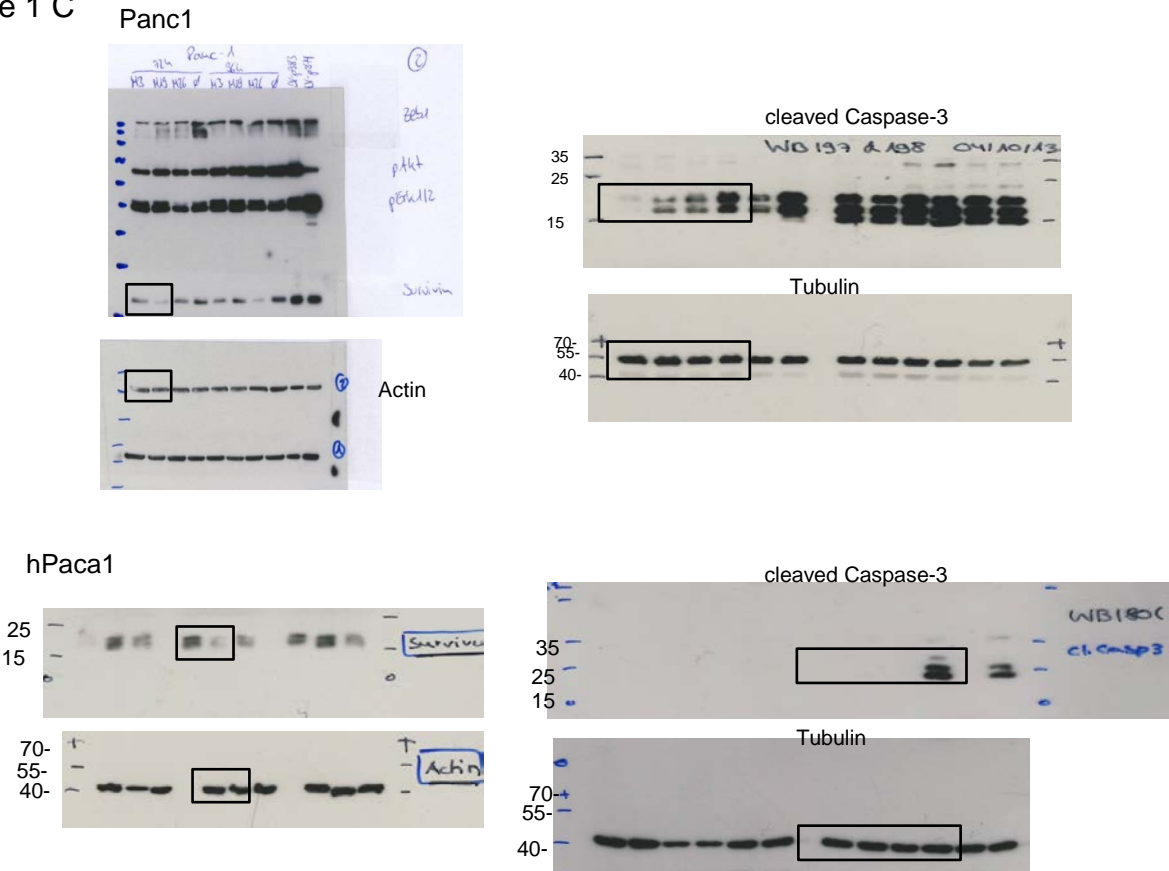

Supplement: Supplementary file 5 [file emmm0007-0831-sd5.pdf]

Figure 3C      Panc1

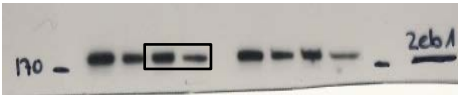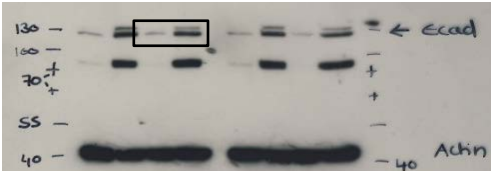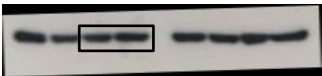

Actin  
Short exp.

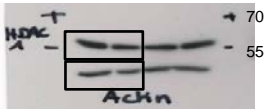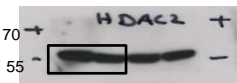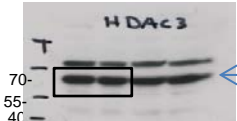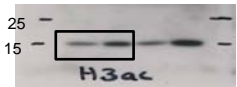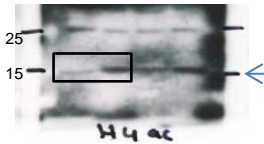

Figure 3E      Panc1

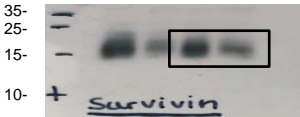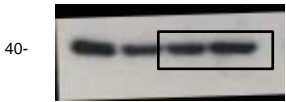

Actin

Supplement: Supplementary file 6 [file emmm0007-0831-sd6.pdf]

Figure 4A

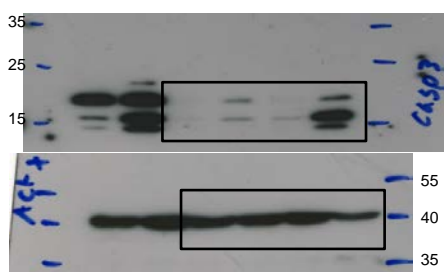

Figure 4C hPaca1 hPaca2

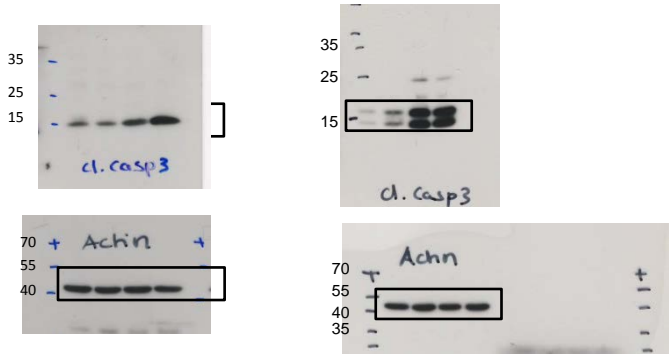

Figure 4D DU-145

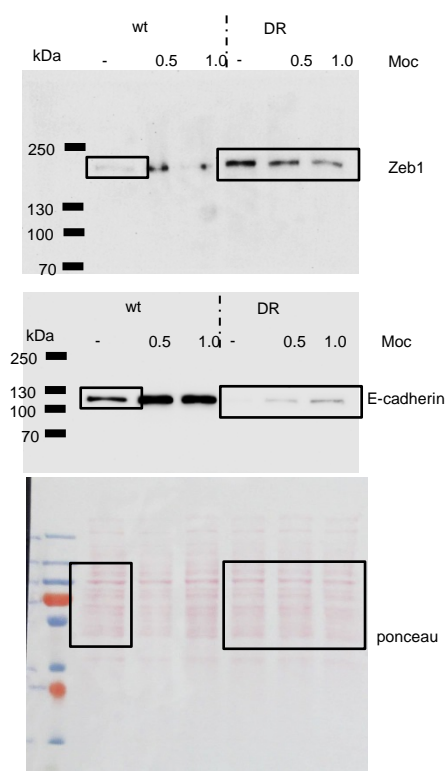

Supplement: Supplementary file 7 [file emmm0007-0831-sd7.pdf]
