# Supplementary material for: ZEB1-associated drug resistance in cancer cells is reversed by the class I HDAC inhibitor mocetinostat
Source: EMBO Mol Med. 2015 Apr 14;7(6):831–47. doi: 10.15252/emmm.201404396 (PMC4459821; doi:10.15252/emmm.201404396)
Supplement: Supplementary file 4 [file emmm0007-0831-sd4.pdf]

EMBO Molecular Medicine

ZEB1-associated drug resistance in cancer cells is reversed by the class I HDAC-inhibitor mocetinostat

Simone Meidhof, Simone Brabletz, Waltraut Lehmann, Bogdan-Tiberius Preca, Kerstin Mock, Manuel Ruh, Julia Schüler, Maria Berthold, Anika Weber, Ulrike Burk, Michael Lübbert, Martin Puhr, Zoran Culig, Ulrich Wellner, Tobias Keck, Peter Bronsert, Simon Küsters, Ulrich T. Hopt, Marc P. Stemmler and Thomas Brabletz

Corresponding author: Thomas Brabletz, Univiversity of Erlangen

Review timeline:

Submission date:	3 July 2014
Editorial Decision:	06 August 2014
Revision received:	04 February 2015
Editorial Decision:	25 February 2015
Revision received:	09 March 2015
Accepted:	16 March 2015

Transaction Report:

(Note: With the exception of the correction of typographical or spelling errors that could be a source of ambiguity, letters and reports are not edited. The original formatting of letters and referee reports may not be reflected in this compilation.)

Editor: Roberto Buccione

1st Editorial Decision 06 August 2014

Thank you for the submission of your manuscript to EMBO Molecular Medicine. We have now heard back from the three Reviewers whom we asked to evaluate your manuscript.

You will see that the Reviewers are mostly supportive of you work, although all they do mention a number of partly overlapping issues with varying degrees of concern. This prevents us from considering publication at this stage. I will not dwell into much detail, as the evaluations are self-explanatory. I would like, however, to highlight a few main points.

Reviewer 1, while acknowledging the potential interest of your work, provides a detailed and critical assessment. The main issues are related to certain inconsistencies and insufficient experimental support for some claims. The Reviewer notes that data interpretation and assessment of significance of the epigenetic drug screening are t arduous and suggests a number of standard approaches to clarify the issue. S/he also notes inconsistencies in the data obtained from PD cell lines. Reviewer 1 would also like to see additional mechanistic insight with respect to how mocetinostat sensitises to gemcitabine and specifically conclusive evidence that this is via ZEB1-mediated chemoresistance. Also listed are other instances of poorly presented and/or difficult to interpret data.

Reviewer 2 is also positive but raises important points. First, s/he notes that despite the claim made, the only drug actually tested to assess mocetinostat's sensitising action was gemcitabine, and thus asks whether other chemotherapics of relevance would fit into the same scenario. The Reviewer also notes, similarly to Reviewer 1, that the miR data do not appear to be conclusive.

Reviewer 3 is largely positive but laments the confusing nature of the presentation and the data overload and notes some incomplete statistical analysis. You will note that Reviewer 1 raised some of the same issues. I would encourage you to undertake a significant effort to streamline the manuscript to make it more approachable to a larger audience. I would not necessarily see the need to omit data as suggested, provided such streamlining is successfully implemented.

Considered all the above, while publication of the paper cannot be considered at this stage, we would be pleased to consider a revised submission, with the understanding that the Reviewers' concerns must be addressed as outlined above, with additional experimental data where appropriate and that acceptance of the manuscript will entail a second round of review.

I look forward to seeing a revised form of your manuscript as soon as possible.

***** Reviewer's comments *****

Referee #1 (Comments on Novelty/Model System):

Statistics and data presentation need to be improved. Some clarifications are also needed

Referee #1 (Remarks):

Zeb1 is an EMT-TF (Epithelial to Mesenchymal Transition Transcription Factor) key during embryonic development and in tumour initiation and different steps of the metastatic cascade. In addition, their activation has been associated with resistance to therapy due to the acquisition of a stem cell phenotype. In particular the same authors showed that Zeb1 promotes tumourigenesis by suppressing microRNAs of the miR200 family that inhibit stemness (Wellner et al. 2009). In the manuscript under consideration, Meidhof and colleagues show that miR-203 in particular, confers drug sensitivity to ZEB1-expressing chemotherapy resistant cancer cells and investigated ZEB1-dependent epigenetic modifications on miR-200, miR-203 and E-Cadherin. Using increased miR-203 levels as readout, the authors screened for drugs able to interfere with ZEB1 function, including ZEB1-dependent epigenetic modifications. Finally, the HDACi mocetinostat was selected as the main target of the screen and validated in vitro and in xenograft models.
The manuscript is interesting and the results have a clear potential therapeutic use but the study contains some inconsistencies and some of the conclusions are not well presented and/or substantiated by the data.

General comments

1. One of the key points of the manuscript is a screening for epigenetics drugs to cooperate with chemotherapy which can decrease cell survival or proliferation by inducing the differentiation of resistant cells to a stemness phenotype. Unfortunately, it is very difficult to interpret the data and the significance of the results as they are presented. Standard measurement of the effects and potency of the chemotherapy agents and the different combinations used should be shown included for the different cell lines (i.e. EC50,half maximal effective concentration). In addition, do the epigenetic drugs show a synergistic/additive effect with the chemotherapy agents?

2. It is valuable to show results from patient-derived cell lines but some of the conclusions are not supported by the data and there are important inconsistencies that the authors should address.
For instance, according to the results shown in Fig 1a, hPACA1 cells (high levels of ZEB1, low miR-200, 203 etc) are much more sensitive to gemcitabine than hPACA2 cells (low levels of ZEB1 and high of the miRs and cadherin) (see scale of the concentrations and again EC50 values should have been presented to compare). Therefore, the assumption made that ZEB1 expression and downregulation of the target miRS promotes chemoresistance in these cells is not sustained by those data. Results from Supp Fig 6b further confirm that hPACA1 cells are more sensitive to gemcitabine than hPACA 2 cells (% cell viability in cells treated with gemcitabine 5 nm). This should be clarified and discussed.
In addition, in Figure 1a; what is the EC50 for gemcitabine in hPACA1 cells? That cannot be calculated from the graph since the authors have not used a wide enough range of concentrations. It seems that it is in the 8-10 nm range (pretty low) and therefore an EC50 of approximately 5 nm in

the gemcitabine+miRs combination does not seem a significant enough shift. How was the statistic test performed in this figure?
Furthermore, Supp Fig 3 (Related to Fig 1) is very confusing- What does negative cell viability means in biological terms? In Supp Fig 3b the authors present a percentage of cell viability in hPaca2 cells after treatment with gemcitabine totally different from Fig 1b. These results seem to be inconsistent. In addition, Supp Fig 3d and 3e are repeats of Fig 1 a and b. Again, the change in cell viability for the combination gemcitabine+miRS or gemcitabine+ anta-miRS does not seem to be relevant o even significant as shown in Supp Fig 3D.

3. The authors show that Zeb1 promotes certain epigenetic changes in the E-Cadherin, miR-200 and miR-203 promoters and performed a screening for epigenetic drugs to interfere with ZEB1 function by using increased miR-203 levels as a readout. However, given the inconsistencies and not very clear results obtained with the patient-derived cell lines hPACA1 and 2 and although is clear that mocetinostat sensitizes cells to gemcitabine treatment, additional mechanistic insight is needed. Does depletion of ZEB1 and/or overexpression of miR-203 in Panc1 or hPACA1 cells block mocetinostat-driven gemcitabine sensitivity in these cells? In other words, can the authors prove that mocetinostat reverses ZEB1-driven chemoresistance as stated in the title of the manuscript?

4. Regarding the in vivo experiments presented in Figure 5 and Supp Fig 6, once again there are some inconsistencies with hPACA cells. Why mice harbouring hPACA1 tumours are treated with 120 mg/kg/day of gemcitabine instead of 25 mg/kg/day as Panc1 and hPACA2 tumour-bearing mice? Results are difficult to interpret without additional clarifications. Furthermore, both immunohistochemistry and in situ hybridization panels are of poor quality. Higher magnifications of the photomicrographs and scale bars need to be presented.

Minor points

1. Some of the results are not very carefully presented. For instance in Suppl Fig 1, absorbance at 570 is presented instead of % cell viability.
2. Some of the statements made in the text need to be further demonstrated. For example, the authors claim that ZEB1 is a "crucial determinant for mediating resistance to chemotherapeutics as well as targeted drugs in different cancer types". However, Supp Fig 1D shows that ZEB1 depletion in A549 tarceva-resistant cells does not have a significant effect in cell viability.
3. The authors also claim that miR-203 in Fig 1G or mocetinostat in Fig 3 promote autophagy. However, only LC3BII levels detected by WB are shown. In addition, the relevance of autophagy in this context is not clear For example, it has been recently shown that LC3BII can be regulated by HDAC6 (Lu KP et al, BBRC 2013).
All in all, in addition to paying attention to specific points, the manuscript needs editing and a bit more careful discussion and interpretation of the actual data.

Referee #2 (Remarks):

This manuscript by Meidhof et al. examines the role of the EMT-activator ZEB1, and its target gene miR-203, in modulating resistance to the chemotherapeutic gemcitabine in pancreatic cancer. The authors conclude from their studies that high Zeb2 and low miR-203 expression levels confer resistance to gemcitabine. This resistance can be attenuated by treatment with the HDAC inhibitor mocetinostat, which reduces Zeb1 levels and restores miR-203 expression.

Major concerns:

The manuscript title "ZEB1-mediated drug resistance in cancer cells is reversed by the class I HDAC-inhibitor mocetinostat" implies that the role of mocetinostat in reversing resistance against multiple chemotherapeutic drugs was assessed in this study. Instead, mocetinostat's function as a drug sensitizer was only assessed using the nucleoside analog gemcitabine. Would a similar reversal of drug resistance be obtained with treatment of Paclitaxel or Tarceva resistant cells (suppl. Figure 1) with mocetinostat?

The authors states on page 5 (results section) and in Figure 1B and suppl. Figure 3E that inhibition of either miR-203 or all miR-200 members using antagomirs increased gemcitabine resistance in hPaca2 cells. This conclusion is not clear from the data presented, there appears to be no difference in cell viability upon miRNA inhibition in the two independent experiments shown.

Figure 1D: The authors state that the role of miR-203 as an inducer of drug sensitivity may be due to its function in cancer stem cell maintenance. Mir-203 expression in Panc1 reduced CD24 and CD44 cell surface expression; however miR-203 expression had no effect on hPaca1 CD44/CD24 levels. Despite these differences, a decrease in sphere formation was observed in both cell lines. Additional, markers of cancer cell stemness (e.g. ESA, ALDH1) would be useful in this analysis.

Minor concerns/comments:

In the material and methods section, the authors state that the quantification of sphere formation was different between Panc1 and hPaca1. Colonies with a diameter greater than 75uM for Panc1 and greater 30uM for hPaca1 cells were counted as spheres, this difference in quantification should be stated in Figure 1.

Poor quality of mirna isH images in Suppl. Figure 7b

Typo-page 9 (discussion), line18, replace 'surviving' with 'survivin'

Type-page 13 (Materials and methods) line 5 replace 'hPac1' with 'hPaca1'

Referee #3 (Remarks):

The following manuscript entitled: "ZEB1-mediated drug resistance in cancer cells is reversed by the class I HDAC-inhibitor mocetinostat" details the identification and evaluation of the class I HDAC-inhibitor, mocetinostat, as a chemosensitizer to gemcitabine, mainly in pancreatic cancer cell lines. Using standard pancreatic cancer cell lines (Panc1 and BxPC3) and two patient-derived cell lines, the authors show that the EMT-activator ZEB1 was important for resistance to the chemotherapeutic drug, gemcitabine. The current work seems to be a continuation of their previous studies involving ZEB1 in cancer and EMT. The authors presently demonstrate that miR-203, along with the previously examined miR-200, is negatively regulated by ZEB1 and was found to be under-expressed in undifferentiated and/or drug-resistant cells. The authors describe a systematic approach to interfere with ZEB1 function and restore sensitivity to chemotherapeutic drugs. Screening for epigenetic drugs, the authors find that treatment with HDAC inhibitor mocetinostat could decrease levels of ZEB1 and increased levels of miR-203.
While the work found in this manuscript is very interesting and medically important - as pancreatic cancer has a dismal survival rate - the manuscript is very dense and hard to read. It took several readings by the reviewer to grasp the full experimental thrust and their salient findings. The work is data heavy with six figures and seven supplemental figures. Sticking just with pancreatic cancer cell lines, and avoiding other cell types, would make it easier to read. The first section entitled: "ZEB1 confers resistance to various drugs in different cancer types" could be left out, as other cell types and previously published findings described seem to detract from the flow. For example, three supplemental figures are introduced before the authors actually get to Figure 1. Some data provided seems superfluous and to slow down the flow, such as sphere forming capacity (Fig 1E) and autophagy induction (Fig 1G). Some statistical analysis seems to be missing, such as drug studies found in Supplemental Figure 4 and histone mark analysis found in Supplemental Figure 5.
While the current work is very interesting and valuable, the current state of the manuscript detracts from their findings. Trimming down the data overload and making the text easier to read would greatly enhance the reader's grasp of the findings.

1st Revision - authors' response 04 February 2015

Revision of manuscript EMM-2014-04396-T

Reply to editor's comments

Thank you for the submission of your manuscript to EMBO Molecular Medicine. We have now heard back from the three Reviewers whom we asked to evaluate your manuscript.

You will see that the Reviewers are mostly supportive of you work, although all they do mention a number of partly overlapping issues with varying degrees of concern. This prevents us from considering publication at this stage. I will not dwell into much detail, as the evaluations are self-explanatory. I would like, however, to highlight a few main points

.

Reviewer 1, while acknowledging the potential interest of your work, provides a detailed and critical assessment. The main issues are related to certain inconsistencies and insufficient experimental support for some claims. The Reviewer notes that data interpretation and assessment of significance of the epigenetic drug screening are t arduous and suggests a number of standard approaches to clarify the issue. S/he also notes inconsistencies in the data obtained from PD cell lines. Reviewer 1 would also like to see additional mechanistic insight with respect to how mocetinostat sensitises to gemcitabine and specifically conclusive evidence that this is via ZEB1-mediated chemoresistance. Also listed are other instances of poorly presented and/or difficult to interpret data.

The comments and suggestions of reviewer 1 were extremely helpful. We addressed all queries and performed all requested experiments. In particular we repeated all drug assays including higher doses, which allowed the determination of EC50 and EC80 doses and subsequent calculations of potential synergistic effects of drug combinations using standard procedures.
We also repeated all experiments with the patient derived cell lines und could explain and resolve all inconsistences criticized by the reviewer (see also reply to his point 2).
As suggested we also addressed the question of a mechanistic link between the drug sensitizing effects of mocetinostat and the downregulation of ZEB1 and upregulation of target microRNAs. In all cellular systems an artificial overexpression of ZEB1 resulted in a decreased cell proliferation, which then was no more compatible for testing an effect on drug combinations (mocetinostat and gemcitabine) in MTT-assays. Therefore we put more effort for the revision work to inhibit the mocetinostat-mediated upregulation of miR-203 and miR-200 in Panc1 by antagomirs and determine if this would reduce the drug-sensitizing effect of mocetinostat. We saw that combined inhibition of miR-203 and miR-200 in mocetinostat and gemcitabine treated cells led to an increase in MTT-activity, which would potentially fit to our hypothesis. However the result was not informative, because antagomir treatment also increased MTT-activity of gemcitabine single treated Panc1 cells (new suppl. Fig. E3D). Therefore we can not make an unambiguous statement concerning the effect of mocetinostat and discussed this aspect in the discussion section.
We also streamlined the paper and weakened some of the conclusions as suggested.

Reviewer 2 is also positive but raises important points. First, s/he notes that despite the claim made, the only drug actually tested to assess mocetinostat's sensitising action was gemcitabine, and thus asks whether other chemotherapics of relevance would fit into the same scenario. The Reviewer also notes, similarly to Reviewer 1, that the miR data do not appear to be conclusive.

Again we could successfully address and resolve all queries of this reviewer. In particular we included another cancer type (the prostate cancer cell line DU 145 and the drug resistant subclone DU145 DR) and another chemotherapeutic (the taxol-derivate docetaxel). We could validate the data generated in the pancreatic cell lines: the drug resistant subclone increased expression of ZEB1 and decreased miR-200 and miR-203. Mocetinostat treatment partially reversed the phenotype (downregulated ZEB1 expression and upregulated miR-203 and miR-200) and also enhanced

sensitivity to docetaxel in a synergistic manner (see new Fig. 1A, new Fig. 4D, new Table 1 and new Table 2).
We also resolved his critics to the effects of miR-203 in patient derived cell lines, particularly also by determinting the changes at the EC50 and EC80 level (which was suggested by reviewer 1).

Reviewer 3 is largely positive but laments the confusing nature of the presentation and the data overload and notes some incomplete statistical analysis. You will note that Reviewer 1 raised some of the same issues. I would encourage you to undertake a significant effort to streamline the manuscript to make it more approachable to a larger audience. I would not necessarily see the need to omit data as suggested, provided such streamlining is successfully implemented.

We streamlined the paper and as suggested by the reviewer deleted many supplemental data to reduce the criticized data overload. This results in the reduction of supplementary Figures from 7 to 4. We did not omit many cell data (particularly did not focus on pancreatic cancer) as suggested, but instead followed the other reviewers and included data, particularly another cell type (prostate and docetaxel). Despite including the requested additional data, we were able to significantly shorten the overall data load.
However the reviewer requested to include all statistical data of our drug screen. In the interest of not overloading the figures again, we summarized it in the Table E1.

Considered all the above, while publication of the paper cannot be considered at this stage, we would be pleased to consider a revised submission, with the understanding that the Reviewers' concerns must be addressed as outlined above, with additional experimental data where appropriate and that acceptance of the manuscript will entail a second round of review.

Please note that it is EMBO Molecular Medicine policy to allow a single round of revision only and that, therefore, acceptance or rejection of the manuscript will depend on the completeness of your responses included in the next, final version of the manuscript.

Revision of manuscript EMM-2014-04396-T

Reply to Referee #1:

We are grateful for the very helpful comments. Based the suggestions we could improve the manuscript and included additional data, informations, and new figures. Please see our point by point reply below your specific comments:

Zeb1 is an EMT-TF (Epithelial to Mesenchymal Transition Transcription Factor) key during embryonic development and in tumour initiation and different steps of the metastatic cascade. In addition, their activation has been associated with resistance to therapy due to the acquisition of a stem cell phenotype. In particular the same authors showed that Zeb1 promotes tumourigenesis by suppressing microRNAs of the miR200 family that inhibit stemness (Wellner et al. 2009). In the manuscript under consideration, Meidhof and colleagues show that miR-203 in particular, confers drug sensitivity to ZEB1-expressing chemotherapy resistant cancer cells and investigated ZEB1-dependent epigenetic modifications on miR-200, miR-203 and E-Cadherin. Using increased miR-203 levels as readout, the authors screened for drugs able to interfere with ZEB1 function, including ZEB1-dependent epigenetic modifications. Finally, the HDACi mocetinostat was selected as the main target of the screen and validated in vitro and in xenograft models.
The manuscript is interesting and the results have a clear potential therapeutic use but the study contains some inconsistencies and some of the conclusions are not well presented and/or substantiated by the data.

General comments:

1. One of the key points of the manuscript is a screening for epigenetics drugs to cooperate with chemotherapy which can decrease cell survival or proliferation by inducing the differentiation of

resistant cells to a stemness phenotype. Unfortunately, it is very difficult to interpret the data and the significance of the results as they are presented. Standard measurement of the effects and potency of the chemotherapy agents and the different combinations used should be shown included for the different cell lines (i.e. EC50,half maximal effective concentration). In addition, do the epigenetic drugs show a synergistic/additive effect with the chemotherapy agents?

This was a very helpful suggestion, which we think led to a strongly increased significance of our data. As suggested we repeated all drug assays including higher doses, which allowed the determination of EC50 and EC80 doses and subsequent calculations of potential synergistic effects of drug combinations. We substituted all old graphs with new ones (now new Fig. 1B,D; Fig. 4B,C; suppl. Fig. E1A,B; suppl. Fig. E2C) and included two tables for listing the EC50 shifts induced by microRNAs (new Table 1) and the calculations for synergistic effects of drug combinations (new Table 2). In addition, by determination of the EC50 and drug synergy, we also validated the drug sensitizing effects of mocetinostat in a different type of cancer and different chemotherapeutic, the treatment of prostate cancer with the taxol-derivate docetaxel (new Fig. 4D).

2. It is valuable to show results from patient-derived cell lines but some of the conclusions are not supported by the data and there are important inconsistencies that the authors should address.

We fully agree about the inconsistencies and invested a large time of the revision process to improve the data concerning the two patient-derived cell lines (see reply to specific comments below).
We wanted to validate the initial data from cell lines and include cell lines directly derived from patients tumors for validation, because they are more close to real situation in patients than established cell lines selected for years. However one disadvantage in working with such cells is, that they are not characterized, might possess different genetic alterations, are still a heterogenous mix of cells from the primary tumors and at the beginning are not highly consistent in repeated experiments. However we solved some of the initial problems with culture conditions and could resolve many inconsistencies.

For instance, according to the results shown in Fig 1a, hPACA1 cells (high levels of ZEB1, low miR-200, 203 etc) are much more sensitive to gemcitabine than hPACA2 cells (low levels of ZEB1 and high of the miRs and cadherin) (see scale of the concentrations and again EC50 values should have been presented to compare). Therefore, the assumption made that ZEB1 expression and downregulation of the target miRs promotes chemoresistance in these cells is not sustained by those data. Results from Supp Fig 6b further confirm that hPACA1 cells are more sensitive to gemcitabine than hPACA 2 cells (% cell viability in cells treated with gemcitabine 5 nm). This should be clarified and discussed.
In addition, in Figure 1a; what is the EC50 for gemcitabine in hPACA1 cells? That cannot be calculated from the graph since the authors have not used a wide enough range of concentrations. It seems that it is in the 8-10 nm range (pretty low) and therefore an EC50 of approximately 5 nm in the gemcitabine+miRs combination does not seem a significant enough shift. How was the statistic test performed in this figure?

Again the suggestion to determine EC50 values was very helpful. We repeated all experiments with higher drug doses. The cells now behaved more consistent and we could show that hPaca1 and hPaca2 have similar EC50 *in vitro*, with the Zeb1 expressing line hPaca 1 beeing slightly more resistant (new suppl. Fig. E2C and Table 1). (However, *in vivo* hPaca1 was much more resistent to gemcitabine than hPaca2, see comment to point 4). Your suggestions allowed also to determine that the effects of miR-203 on gemcitabine resistance in hPaca 1 were more prominent at the EC80 compared to the EC50 level (new Fig. 1B and Table1). We applied the two-way ANOVA with Tukey's multiple comparisons test for MTT-assays. Also the effects of antagomirs on endogenous miR-203 and miR-200 (increase of resistance) in hPaca2 were more significant at the EC80 level (new Fig. 1D). We think that the more prominent effect at the EC80 level in patient-derived cell lines might reflect the heterogenous population in such lines: sensitive subpopulation die early, but resistant subpupulations survive lower doses. These resistant subpopulations might be sensitized by miR-203 and can now also be targeted by gemcitabine at medium doses (shift in EC80). In fact such a behavior could better reflect the situation in the patient than using long-established cell lines. Also in patients resistant subclones often survive chemotherapy and are the source of disease relapse.
We now also completely repeated the drug combination assays and calculated the synergy effects based on EC50 values. The data were now again much more consistant and showed a statistically

synergistic effect for combining mocetinostat and gemcitabine in Panc1 and hPaca1, but no synergism in hPaca2 (new Fig. 4 B, C and Table 2).
In summary: both patient derived lines have a similar susceptibility to gemcitabine, although hPaca1 expresses high ZEB1. This for instance might be due to unknown differences in underlying genetic alterations, etc. However we could confirm in these two patient derived lines that high ZEB1 levels predicted an increase in the sensitivity to chemotherapy by a combination with mocetinostat, which was not detected in hPaca2 with low ZEB1.

Furthermore, Supp Fig 3 (Related to Fig 1) is very confusing- What does negative cell viability means in biological terms? In Supp Fig 3b the authors present a percentage of cell viability in hPaca2 cells after treatment with gemcitabine totally different from Fig 1b. These results seem to be inconsistent. In addition, Supp Fig 3d and 3e are repeats of Fig 1 a and b. Again, the change in cell viability for the combination gemcitabine+miRS or gemcitabine+ anta-miRS does not seem to be relevant o even significant as shown in Supp Fig 3D.

As explained above, we solved the problems with inconsistent repetitions. The repeated experiments wer now very constistent, could be included in one graph (new Fig. 1 B and D) and therefore the old suppl. Fig. 3D and E could be deleted.
In deed we agree that the term "negative cell viability" was confusing and misleading. We substituted it by "relative MTT-activity" in all MTT-assays (see new Methods section: … Relative MTT activity was then calculated relative to activity one day after seeding (set to 0%). The activity of untreated cells 72 hours after starting of drug treatment was set to 100%. A negative activity means that the drug reduced cell number below the number of cells at treatment start…).

3. The authors show that Zeb1 promotes certain epigenetic changes in the E-Cadherin, miR-200 and miR-203 promoters and performed a screening for epigenetic drugs to interfere with ZEB1 function by using increased miR-203 levels as a readout. However, given the inconsistencies and not very clear results obtained with the patient-derived cell lines hPACA1 and 2 and although is clear that mocetinostat sensitizes cells to gemcitabine treatment, additional mechanistic insight is needed. Does depletion of ZEB1 and/or overexpression of miR-203 in Panc1 or hPACA1 cells block mocetinostat-driven gemcitabine sensitivity in these cells? In other words, can the authors prove that mocetinostat reverses ZEB1-driven chemoresistance as stated in the title of the manuscript?

We agree to the vers straight-forward comments and already tried to further increase mechanistic links between the drug-sensitizing effects of mocetinostat and the demonstrated effects of ZEB1 and it targets microRNAs. In fact we tried to perform exactly the experiments suggested by the reviewer to be included in the first manuscript version. However we faced conceptual and technical problems. In all cellular systems an artificial overexpression of ZEB1 resulted in a decreased cell proliferation, which then was no more compatible for testing an effect on drug combinations (mocetinostat and gemcitabine) in MTT-assays. Therefore we put more effort for the revision work to inhibit the mocetinostat-mediated upregulation of miR-203 and miR-200 in Panc1 by antagomirs and determine if this would reduce the drug-sensitizing effect of mocetinostat. We saw that combined inhibition of miR-203 and miR-200 in mocetinostat and gemcitabine treated cells led to an increase in MTT-activity, which would potentially fit to our hypothesis. However the result was not informative, because antagomir treatment also increased MTT-activity of gemcitabine single treated Panc1 cells (new suppl. Fig. E3D). Therefore we can not make an unambiguous statement concerning the effect of mocetinostat and discussed this aspect in the discussion section.
Anyhow, we did not want to claim that upregulation of miR-203/200 and inhibition of ZEB1 are the only or major molecular effects of mocetinostat. As an HDACinhibitor it might have many other, still unknown molecular effects and target genes, which explain its efficiency in restoring drug sensitivity. Due to the current lack of a direct molecular proof, we also discussed this aspect in the discussion section and weakened the statements concerning the role of ZEB1, miR-200 and miR-203 in mediating the effect of mocetinostat all over the manuscript and made more carefull discussion statements and interpretations of the actual data, as suggested by the reviewer. For instance we already changed the title to "ZEB1-associated…" instead of "ZEB1-mediated…."
However, as demonstrated by our work the usage of miR-203 and miR-200 upregulation as readout turned out to be a good indicator and very useful tool for our initial small scale drug screen and might now be applied also for larger screens.

4. Regarding the in vivo experiments presented in Figure 5 and Supp Fig 6, once again there are some inconsistencies with hPACA cells. Why mice harbouring hPACA1 tumours are treated with 120 mg/kg/day of gemcitabine instead of 25 mg/kg/day as Panc1 and hPACA2 tumour-bearing mice? Results are difficult to interpret without additional clarifications.

Before applying combination treatments *in vivo*, we performed single agent treatments to determine the best drug doses and treatment schemes to study the combination effects. Thereby hPaca1 turned out grow very fast and to be much higher resistant to gemcitabine than hPaca2. This was in clear contrast to the *in vitro* data, where both lines show similar sensitivity, but fits to the differences in the expression pattern of ZEB1 and miR-203. We therefore increased the gemcitabine dose for combination studies (but note the also the regiment was changed: hPaca1 high dose but only once a week, hPaca2 low dose but twice a week. This is now also better explained in the legend to new suppl. Fig. E4). Despite the remarkable tumor growth even at higher single gemcitabine dose, a combination with mocetinostat acted synergistically. In contrast (and in line with the in vitro data) there was no synergistic effect in hPaca2.
These data also clearly pointed out to differences of *in vivo* and *in vitro* tests using patient derived cell cultures. They might indicate that *in vivo* experiments reflect better the clinical behavior and situation than *in vitro* assays. Factors could of course be the crosstalk with the environment or niches, which however are not the focus of this study.

Furthermore, both immunohistochemistry and in situ hybridization panels are of poor quality. Higher magnifications of the photomicrographs and scale bars need to be presented.

As suggested, we now improved the quality of immunohistochemistry and in situ hybridization panels and included inserts with higher mangifications and scale bars (new Fig. 5 B,C and new suppl. Fig. E4 B,C,E).

Minor points

1. Some of the results are not very carefully presented. For instance in Suppl Fig 1, absorbance at 570 is presented instead of % cell viability.

The old suppl. Fig. 1 was deleted, as suggested by the other reviewers.

2. Some of the statements made in the text need to be further demonstrated. For example, the authors claim that ZEB1 is a "crucial determinant for mediating resistance to chemotherapeutics as well as targeted drugs in different cancer types". However, Supp Fig 1D shows that ZEB1 depletion in A549 tarceva-resistant cells does not have a significant effect in cell viability.

Old suppl. Fig. 1 including A549 was deleted, as suggested by the other reviewers. Instead another cell line, the prostate cancer cell line DU-145 and its doxetaxel resistant subclone DU145-DR was successfully included in the study (see new Fig. 1A and new Fig. 4D, Table 1 and Table 2).

3. The authors also claim that miR-203 in Fig 1G or mocetinostat in Fig 3 promote autophagy. However, only LC3BII levels detected by WB are shown. In addition, the relevance of autophagy in this context is not clear For example, it has been recently shown that LC3BII can be regulated by HDAC6 (Lu KP et al, BBRC 2013).

In the interest of shortening and streamlining the manuscript, the preliminary data concerning autophagy were deleted.

All in all, in addition to paying attention to specific points, the manuscript needs editing and a bit more careful discussion and interpretation of the actual data.

We fully agree and streamlined the paper, particularly by reducing the supplemental Figures from 7 to 4. Moreover we weakened many statements and interpretations, as suggested (see also answers to specific points above).

Reply to Referee #2:

We are grateful for the very helpful comments. Based the suggestions we could improve the manuscript and included additional data, informations, and new figures. Please see our point by point reply below your specific comments:

Referee #2 (Remarks):

This manuscript by Meidhof et al. examines the role of the EMT-activator ZEB1, and its target gene miR-203, in modulating resistance to the chemotherapeutic gemcitabine in pancreatic cancer. The authors conclude from their studies that high Zeb2 and low miR-203 expression levels confer resistance to gemcitabine. This resistance can be attenuated by treatment with the HDAC inhibitor mocetinostat, which reduces Zeb1 levels and restores miR-203 expression.

Major concerns:

The manuscript title "ZEB1-mediated drug resistance in cancer cells is reversed by the class I HDAC-inhibitor mocetinostat" implies that the role of mocetinostat in reversing resistance against multiple chemotherapeutic drugs was assessed in this study. Instead, mocetinostat's function as a drug sensitizer was only assessed using the nucleoside analog gemcitabine. Would a similar reversal of drug resistance be obtained with treatment of Paclitaxel or Tarceva resistant cells (suppl. Figure 1) with mocetinostat?

We agree to the reviewer that a validation of the effects of mocetinostat in other tumor types and for other chemotherapeutics would increase the relevance of our findings. We now included another cell line, the prostate cancer cell line DU-145, sensitive to the taxol-derivate docetaxel and its drug-resistant subclone DU145-DR. We could validate the data generated in the pancreatic cell lines: the drug resistant subclone increased expression of ZEB1 and decreased miR-200 and miR-203. Mocetinostat treatment partially reversed the phenotype (downregulated ZEB1 expression and upregulated miR-203 and miR-200) and also enhanced sensitivity to docetaxel in a synergistic manner (see new Fig. 1A, new Fig. 4D, new Table 1 and new Table 2).
Unfortunately, we could no more test the Tarceva resistant H358 clones as suggested, because they could not be regrown after the lab moved to another university at the beginning of the revision work.

The authors states on page 5 (results section) and in Figure 1B and suppl. Figure 3E that inhibition of either miR-203 or all miR-200 members using antagomirs increased gemcitabine resistance in hPaca2 cells. This conclusion is not clear from the data presented, there appears to be no difference in cell viability upon miRNA inhibition in the two independent experiments shown.

We fully agree about the inconsistencies and invested a large time of the revision process to improve the data concerning the two patient-derived cell lines (see reply to specific comments below).
We wanted to validate the initial data from cell lines and include cell lines directly derived from patients tumors for validation, because they are more close to real situation in patients than established cell lines selected for years. However on disadvantage in working with such cells is, that they are not characterized, might possess different genetic alterations, are still a heterogenous mix of cells from the primary tumors and at the beginning are not highly consistent in repeated experiments. However we solved some of the initial problems with culture conditions and can now present more consistent data.
For all cell systems and drugs we now determined the EC50 (and sometimes EC80) concentrations. Concerning the effect of antagomirs on hPaca2 we have now more clear data: The effects of antagomirs on endogenous miR-203 and miR-200 (increase of resistance) in hPaca2 was more significant at the EC80 level compared to the EC50 (new Fig. 1D and new Table 1). The same was seen for reciprocal experiment with overexpression of the microRNAs in hPaca1 (new Fig. 1B). We think that the prominent effect at the EC80 level in patient-derived cell lines might reflect the heterogenous population in such lines: sensitive subpopulation die early, but resistant

subpopulations survive lower doses. These resistant subpopulations might be sensitized by miR-203 and can now also be targeted by gemcitabine at medium doses (shift in EC80). In fact such a behavior could better reflect the situation in the patient than using long-established cell lines. Also in patients resistant subclones often survive chemotherapy and are the source of diease relapse.

Figure 1D: The authors state that the role of miR-203 as an inducer of drug sensitivity may be due to its function in cancer stem cell maintenance. Mir-203 expression in Panc1 reduced CD24 and CD44 cell surface expression; however miR-203 expression had no effect on hPaca1 CD44/CD24 levels. Despite these differences, a decrease in sphere formation was observed in both cell lines. Additional, markers of cancer cell stemness (e.g. ESA, ALDH1) would be useful in this analysis.

We agree that compared to Panc1, the effects of miR-203 on the CD44/CD24 positive population was small in hPaca1 compared to Panc1. However this might be due to the fact that this potential cancer stem cell subpopulation is also much smaller in hPaca1 compared to Panc1 (compare FACS staining in new suppl. Fig. E2 B). In this context there is a clear reduction in the double positive population also in hPaca1, mainly due to reduction in CD24 expression (now marked by arrows in new Fig. 1E, comparing black to red). As suggested, we tested further markers of cancer stem cells and also detected a clear reduction in the expression of CD133, another marker described for pancreatic cancer. We further applied the Aldeflour assay for ALDH1 activity, which however did not work in this cell line. In summary we are convinced that miR-203 also affects the potential cancer stem cell fraction in the patient-derived hPaca1.

Minor concerns/comments:

In the material and methods section, the authors state that the quantification of sphere formation was different between Panc1 and hPaca1. Colonies with a diameter greater than 75uM for Panc1 and greater 30uM for hPaca1 cells were counted as spheres, this difference in quantification should be stated in Figure 1.

As suggested, we now stated this also in the legend to new Fig. 1F.

Poor quality of mirna isH images in Suppl. Figure 7b

We now improved the quality of immunohistochemistry and in situ hybridization panels and included inserts with higher mangifications (new Fig. 5 B,C and new suppl. Fig. 4 B,C,E).

Typo-page 9 (discussion), line18, replace 'surviving' with 'survivin'.
Type-page 13 (Materials and methods) line 5 replace 'hPac1' with 'hPaca1'.

Thank you for the carefull reading! We corrected both typos.

Reply to Referee #3:

We are grateful for the very helpful comments. Based the suggestions we could improve the manuscript. In particular we streamlined the manuscript and deleted many supplemental data, which resulted in the reduction from seven to four supplementary figures. Please see our point by point reply below your specific comments:

Referee #3 (Remarks):

The following manuscript entitled: "ZEB1-mediated drug resistance in cancer cells is reversed by the class I HDAC-inhibitor mocetinostat" details the identification and evaluation of the class I HDAC-inhibitor, mocetinostat, as a chemosensitizer to gemcitabine, mainly in pancreatic cancer cell lines. Using standard pancreatic cancer cell lines (Panc1 and BxPC3) and two patient-derived cell lines, the authors show that the EMT-activator ZEB1 was important for resistance to the chemotherapeutic drug, gemcitabine. The current work seems to be a continuation of their previous studies involving ZEB1 in cancer and EMT. The authors presently demonstrate that miR-203, along

with the previously examined miR-200, is negatively regulated by ZEB1 and was found to be under-expressed in undifferentiated and/or drug-resistant cells. The authors describe a systematic approach to interfere with ZEB1 function and restore sensitivity to chemotherapeutic drugs. Screening for epigenetic drugs, the authors find that treatment with HDAC inhibitor mocetinostat could decrease levels of ZEB1 and increased levels of miR-203.

While the work found in this manuscript is very interesting and medically important - as pancreatic cancer has a dismal survival rate - the manuscript is very dense and hard to read. It took several readings by the reviewer to grasp the full experimental thrust and their salient findings. The work is data heavy with six figures and seven supplemental figures. Sticking just with pancreatic cancer cell lines, and avoiding other cell types, would make it easier to read.
The first section entitled: "ZEB1 confers resistance to various drugs in different cancer types" could be left out, as other cell types and previously published findings described seem to detract from the flow. For example, three supplemental figures are introduced before the authors actually get to Figure 1.

Sorry for the difficult to read first version of the manuscript and thank you for staying with the review and giving very helpful suggestions to improve the reading!
As suggested we now left out the first results section and merged a small remaining part with the old second results section. This also allowed us to completely delete the first supplementary Figure and include few of the important pictures in Fig.1, which results in a better reading flow.

Some data provided seems superfluous and to slow down the flow, such as sphere forming capacity (Fig 1E) and autophagy induction (Fig 1G).

As suggested, among many other data all autophagy data were deleted. However, we wanted to keep in the sphere forming assays, because they support an effect of miR-203 and of mocetinostat on potential stemness traits of cancer cells.

Some statistical analysis seems to be missing, such as drug studies found in Supplemental Figure 4 and histone mark analysis found in Supplemental Figure 5.

We now included also all requested statistical analyses. These are for the ChIP data in Fig. 2B,C, Fig. 3D and suppl. Fig. E3B. For the drug studies the inclusion of significance marks directly in the panels of the figures made them unreadable, therefore we included a new supplementary Table 1, which shows the significance of all presented drug studies and refers to the individual pictures.

While the current work is very interesting and valuable, the current state of the manuscript detracts from their findings. Trimming down the data overload and making the text easier to read would greatly enhance the reader's grasp of the findings.

As suggested we streamlined the text and particularly removed a lot of redundant supplementary data, which resulted in the reduction of supplementary Figures from seven to four. This could be done despite the inclusion of additional new data and pictures, which were requested by the other reviewers.

2nd Editorial Decision 25 February 2015

Thank you for the submission of your revised manuscript to EMBO Molecular Medicine. We have now received the enclosed reports from the referees that were asked to re-assess it. As you will see the reviewers are now globally supportive and I am pleased to inform you that we will be able to accept your manuscript pending the following final amendments:

1) As suggested by Reviewer 1, please correct inconsistencies in manuscript layout and for textual redundancy. This Reviewer also mentions improvements that are need for the figures, but please see my point 4 below for a detailed series of requests concerning the figures.

2) As per our Author Guidelines, the description of all reported data that includes statistical testing must state the name of the statistical test used to generate error bars and P values, the number (n) of independent experiments underlying each data point (not replicate measures of one sample), and the actual P value for each test (not merely 'significant' or 'P < 0.05').

3) Data described in submitted manuscripts should be deposited in a MIAME-compliant format with one of the public databases. We would therefore ask you to submit your microarray data to the ArrayExpress database maintained by the European Bioinformatics Institute for example. ArrayExpress allows authors to submit their data to a confidential section of the database, where they can be put on hold until the time of publication of the corresponding manuscript. Please see http:www.ebi.ac.uk/arrayexpress/Submissions/ or contact the support team at arrayexpress@ebi.ac.uk for further information.

4) There are some issues with your figures that require remediation. I note (as did Reviewer 1) that some western blot images have excess contrasting. Please improve them in this respect. Also, image panels in Fig. 4C appear to be missing scale bars. Please indicate on the IHCs in Figure 5B and C wherefrom the insets were magnified in the lower magnification frame (e.g. with an overlayed box). Please try to improve the quality (especially text) of Figure 6: you will see that the text/line art appears blocky/blurry when zooming in. Finally, please be reminded that as our guidelines state "Images gathered at different times or from different locations should not be combined into a single image... If juxtaposing images is essential, the borders should be clearly demarcated in the figure and described in the legend." I note that this may have occurred in your manuscript. Please remedy as per our guidelines and provide the corresponding source data for our perusal.

5) We are now encouraging the publication of source data, particularly for electrophoretic gels and blots, with the aim of making primary data more accessible and transparent to the reader. Would you be willing to provide a PDF file per figure that contains the original, uncropped and unprocessed scans of all or at least the key gels used in the manuscript? The PDF files should be labeled with the appropriate figure/panel number, and should have molecular weight markers; further annotation may be useful but is not essential. The PDF files will be published online with the article as supplementary "Source Data" files. If you have any questions regarding this just contact me.

6) Every published paper now includes a 'Synopsis' to further enhance discoverability. Synopses are displayed on the journal webpage and are freely accessible to all readers. They include a short standfirst (to be written by the editor) as well as 2-5 one sentence bullet points that summarise the paper (to be written by the author). Please provide the short list of bullet points that summarise the key NEW findings. The bullet points should be designed to be complementary to the abstract - i.e. not repeat the same text. We encourage inclusion of key acronyms and quantitative information. Please use the passive voice. Please attach these in a separate file or send them by email, we will incorporate them accordingly.

7) Please remove the red lettering in the manuscript file as it will be no longer needed.

Please submit your revised manuscript within two weeks. I look forward to seeing a revised form of your manuscript as soon as possible.

2nd Revision - authors' response 09 March 2015

Please find the submission of the revised version (V3) of our manuscript EMM-2014-04396 entitled: ZEB1-associated drug resistance in cancer cells is reversed by the class I HDACinhibitor mocetinostat
by Simone Meidhof, Simone Brabletz, Waltraut Lehmann, Bogdan-Tiberius Preca, Kerstin Mock, Manuel Ruh, Julia Schüler, Maria Berthold, Anika Weber, Ulrike Burk, Michael Lübbert, Martin Puhr, Zoran Culig, Ulrich Wellner, Tobias Keck, Peter Bronsert, Simon Küsters, Ulrich T. Hopt, Marc P. Stemmler, Simone Brabletz, Thomas Brabletz,

which we would EMBO Molecular Medicine to consider for publication as a Research Article.

As requested we made the following final amendments (to your points 1-7):
1. We corrected textual redundancy and inconsistencies in the figure layout.
2. For each graph, we included the names of the statistical tests, the number of independent experiments and the actual p-values. As discussed by phone, when not feasible to include in the graph, the exact p-values are shown in the new table E4.
3. The microarrays were deposited in the ArrayExpress database (explained in Materials and Methods).
4. We improved the readability (Fig.6), the contrasting and demarcations in the mentioned western blots. We included the missing scale bars and included boxes to indicate the magnified regions (Fig. 5 and E4).
5. We included original western blots as souce data (in Expanded View).
6. We included bullet points for the synopsis.
7. We removed the red lettering.

Thank you again for your support. Looking forward to your decision.
